# Association between wrist-worn free-living accelerometry and hand grip strength in middle-aged and older adults

**DOI:** 10.1007/s40520-024-02757-z

**Published:** 2024-05-08

**Authors:** Colum Crowe, John Barton, Brendan O’Flynn, Salvatore Tedesco

**Affiliations:** Tyndall National Institute, University College Cork, Lee Maltings, Prospect Row, Cork, T12R5CP Ireland

**Keywords:** Accelerometry, Wearable sensors, Older adults, Hand grip strength, Wrist-band devices, Frailty, Pre-frailty

## Abstract

**Introduction:**

Wrist-worn activity monitors have seen widespread adoption in recent times, particularly in young and sport-oriented cohorts, while their usage among older adults has remained relatively low. The main limitations are in regards to the lack of medical insights that current mainstream activity trackers can provide to older subjects. One of the most important research areas under investigation currently is the possibility of extrapolating clinical information from these wearable devices.

**Methods:**

The research question of this study is understanding whether accelerometry data collected for 7-days in free-living environments using a consumer-based wristband device, in conjunction with data-driven machine learning algorithms, is able to predict hand grip strength and possible conditions categorized by hand grip strength in a general population consisting of middle-aged and older adults.

**Results:**

The results of the regression analysis reveal that the performance of the developed models is notably superior to a simple mean-predicting dummy regressor. While the improvement in absolute terms may appear modest, the mean absolute error (6.32 kg for males and 4.53 kg for females) falls within the range considered sufficiently accurate for grip strength estimation. The classification models, instead, excel in categorizing individuals as frail/pre-frail, or healthy, depending on the T-score levels applied for frailty/pre-frailty definition. While cut-off values for frailty vary, the results suggest that the models can moderately detect characteristics associated with frailty (AUC-ROC: 0.70 for males, and 0.76 for females) and viably detect characteristics associated with frailty/pre-frailty (AUC-ROC: 0.86 for males, and 0.87 for females).

**Conclusions:**

The results of this study can enable the adoption of wearable devices as an efficient tool for clinical assessment in older adults with multimorbidities, improving and advancing integrated care, diagnosis and early screening of a number of widespread diseases.

**Supplementary Information:**

The online version contains supplementary material available at 10.1007/s40520-024-02757-z.

## Introduction

The prevalence of consumer wearables has been rapidly increasing in recent times with fitness trackers nowadays becoming popular devices for monitoring physical activity levels, sports performance, and general health status in real time [1]. Although these devices primarily target a younger, more athletic demographic, their applicability to senior citizens has been a focus of researchers’ interest in recent years [2]. In particular, wrist-based devices are considered the most suitable in terms of user acceptability and user-friendliness [2, 3], and their validation in terms of accuracy and reliability in lab- and home-settings has been studied extensively in literature [4–9].

However, despite the exponential increase in consumer wearables, typical consumer wrist-worn devices still focus on basic information, such as step count, distance traveled, physical activity, heart rate, calories, and sleep patterns. Hence, these technologies have not achieved widespread adoption as medical devices [10]. Nevertheless, their potential to provide clinical-level indications in different contexts through the incorporation of machine learning (ML) has been shown in literature [10–12], This is particularly evident in applications related to human motion analysis, rehabilitation and orthopedics, ageing, metabolic system, physiological monitoring, sleep, psychological stress and cognitive function. Moreover, consumer grade wearable devices have also begun to be used in pilot studies and clinical trials such as for drug development, drug dose and frequency adjustments, detection of early sign of Lyme’s disease, and arrhythmia detection [13].

The potential for commercial wearables to deliver clinical-grade insights is anticipated to become increasingly significant in the future considering the ongoing rise in the aging population within Western societies, which in turn is driving the promotion of preventive medicine, self-health monitoring, remote patient monitoring, and telemedicine [14–16].

In very recent years, hand grip strength has been proposed as a biomarker for ageing [17, 18], able to provide indications on the current health status of an individual, as it is correlated with overall strength, upper limb function, bone mineral density, fractures, falls, malnutrition, cognitive impairment, depression, sleep problems, diabetes, multimorbidity, and quality of life. Moreover, hand grip strength is also considered a predictor for future outcomes [19], specifically all-cause and disease-specific mortality, future function, bone mineral density, fractures, cognition and depression, and problems associated with hospitalization.

However, the only method to obtain this measurement is through a hand dynamometer, which is squeezed with maximum isometric effort for about five seconds, in a clinical setting [20]. The substitution of this procedure with continuously worn wearable technology may be useful for older adults living in rural areas, allowing clinicians to obtain this indicator remotely, without asking the patient to come to the hospital, and implement effective preventive medicine measures.

Despite numerous publications having considered the use of wearable sensing for the detection of the hand pose and reach and grasp activities [21, 22], especially for applications such as stroke rehabilitation, rheumatoid arthritis, and Parkinson’s bradykinesia, few studies have specifically investigated the adoption of devices for grip force profiling. For example, force-measurement gloves and custom-made strain gauge and force sensors [23, 24], surface electromyography (EMG) of the forearm muscles [25], inertial measurement units (IMUs) worn on the lower- and upper-back, upper arm, and thigh [26], facial videos and wearable photoplethysmogram (PPG) data [27], and wearable prototypes based on wrist-based active bone-conducted sound sensing [28], have all been linked to hand grip estimation.

However, while the solutions described in [23–28] showed that other technologies could be used as alternatives to hand dynamometers for the estimation of grip forces, most of those works still require the participants to perform tasks involving maximal contraction efforts which may not be always feasible in home settings and for specific cohorts of subjects. Given the correlation between hand grip strength and physical activity [29, 30], it could be assumed that a wrist-based solution based on motion sensing may estimate the hand grip strength of a subject based on the standard physical activity data collected with a consumer-level wearable device over the course of a number of days. It is evident that there remains a need for further investigation into this topic.

The present study has a twofold goal:

- to investigate the feasibility of using 7-days physical activity data collected via a standard consumer-grade wristband, as well as demographics variables and questionnaire data, in conjunction with ML models, for the estimation of hand grip strength in a cohort of middle-aged and older adults;
- to develop ML models which could detect specific medical conditions based on the categorization resulting from hand grip strength.

## Methods

### Participants and preliminary data processing

This study was based on a random sample of 14,161 subjects, between the age of 40 and 71, which participated in the UK Biobank Study [31]. During data collection, participants wore a wrist-worn accelerometer (Axivity AX3, Axivity Ltd, Newcastle, UK [32]) on the dominant hand for seven days, with sampling rate of the raw triaxial accelerometer set at 100 Hz. This investigation received approval by UK Biobank (Application Number: 47,845).

Demographics (e.g., gender, age, weight, height, body mass index - BMI) and clinical information (i.e., medical conditions) were also collected for each participant. Each subject was categorized according to the reported diseases based on the morbidity clusters proposed in [33]. The categories defined were: diabetes, cancer, cardiac diseases, neurodegenerative diseases, respiratory diseases, and musculoskeletal diseases. The lack of any medical condition deemed the subject as “healthy”, while participants with two or more conditions were instead deemed as “multimorbid” [34]. Further information on the participants’ categorization is shown in Appendix 1. A summary of the demographics and clinical information of the participants is presented in Table 1, with the data distributions for both genders for hand grip strength, age, and BMI are shown in Figure A.1 (supplementary material).

**Table 1:** Participants’ personal and clinical characteristics (*N* = 14,161)

Characteristic	Males (*n* = 6,226)	Females (*n* = 7,935)
Hand Grip Strength Left (kg), mean (SD)	38.95 (8.79)	23.02 (6.43)
Hand Grip Strength Right (kg), mean (SD)	41.20 (8.82)	25.31 (6.52)
Age (years), mean (SD)	57.40 (7.91)	56.15 (7.68)
BMI (kg/m2), mean (SD)	27.32 (3.99)	26.26 (4.79)
Weight (kg), mean (SD)	85.02 (13.54)	70.02 (13.26)
Estimated Height (m), mean (SD)	1.76 (0.07)	1.63 (0.06)
Cancer	280	184
Diabetes	378	696
Cardiac Diseases	1212	844
Neurodegenerative Diseases	38	26
Respiratory Diseases	1184	1504
Musculoskeletal Diseases	672	1212
Multimorbidity	1036	1297

The multi-day raw accelerometer data collected for each participant were processed using the GGIR software package (version 1–11.0) [35]. GGIR is an R-package which is among the most well-known validated toolboxes to process multi-day raw accelerometer data for deriving day-by-day physical activity, and sleep features [36–40]. The features extracted were divided into three groups: “personal”, “day”, and “night” metrics. The physical activity and sleep features obtained were used, along with the demographics and clinical information, to build the dataset. Overall, 327 features were gathered for each participant. Further information on those features is available in Appendix 2.

Finally, the left- and right-hand grip strength measurements were averaged to create a label. The continuous label for hand grip strength (expressed in kg) was also converted in a binary classification label using cut-offs as a criterion for frailty/pre-frailty based on sex and BMI. The ratio between cut-offs for frailty of different BMI ranges reported in [41] was used in conjunction with the T-score method from [42] to define two sets of hand grip strength cut-off values: one corresponding to a T-score of -2 and the other a T-score of -1 (Table 2). Those cut-off values were adopted as they are generally associated with a number of medical conditions; for instance, low grip strength is generally associated to T-score < -2 SD [43]. Similarly, osteopenia is associated with T-score < -1 SD and osteoporosis with T-score < -2.5 SD [44, 45].

**Table 2:** Criteria Used to Define Frailty/Pre-Frailty

	BMI Range	Cutoff for grip strength (kg) criterion for pre-frailty using a T-score of -2	Cutoff for grip strength (kg) criterion for pre-frailty using a T-score of -1
Men	≤ 24	≤ 31.0	≤ 40.6
	24.1–26	≤ 32.1	≤ 42.0
	26.1–28	≤ 32.1	≤ 42.0
	> 28	≤ 34.2	≤ 44.8
Women	≤ 23	≤ 18.9	≤ 24.9
	23.1–26	≤ 19.2	≤ 25.3
	26.1–29	≤ 19.9	≤ 26.3
	> 29	≤ 23.3	≤ 30.7

### Modelling

#### Regression task

For model building, the dataset was separated into male/female subsets with analysis performed independently for both genders, each of which was randomly split into training (4,980/6,348 subjects), validation (996/1,270 subjects) and test sets (1,246/1,587 subjects), accounting for approximately 60%, 20%, and 20% of the total sample size, respectively. Outliers were imputed using the Median Absolute Deviation (MAD) method. The median and MAD for each feature was calculated, values considered outliers based on a threshold value of 2 were replaced with the median value of the training data for that particular feature. Additionally, all the features were standardized before feature selection.

The features were fed into a supervised-based regressor developed in Python 3 (Python Software Foundation, Delaware, US). Regressors considered in this analysis were linear models (Linear Regression, ElasticNet, LASSO, Ridge, BayesianRidge, and RidgeCV regression), decision tree (DT), random forest (RF), and XGBoost. Mean absolute error (MAE) was used as the metric to quantify the goodness-of-fit comparing the predictions of the regressor with the real measurements. A grid search was employed on the training set to attain optimal values for the model hyper-parameters. Model fitting and feature selection (based on Select Percentile using f_regression as a scoring function) were deployed simultaneously. For each combination of hyper-parameters’ values, a 5-fold cross-validation was carried out on the training data and the related MAE was obtained. The combination of hyper-parameters that returned the lowest MAE was considered as the optimum and the selected model was evaluated on the validation set to prove its generalizability. Consecutively, training and validation sets were merged into a single new training set (5,976/7,618 subjects) and the model was re-trained (with the optimal hyper-parameters and features selected) and MAE and standard deviation (SD) were obtained for the test dataset. The random partitioning of subjects into training, validation, and test sets can have an effect on the results so this procedure was repeated 100 times as a convergence of the MAE was observed within this time. Finally, the average and standard deviation of the MAE across all iterations is reported and the parameters selected over the iterations shown in the results.

#### Classification task

Similarly, the dataset was separated into male/female subsets, each of which was randomly split into training (4,980/6,348 subjects), validation (996/1,270 subjects) and test sets (1,246/1,587 subjects), accounting for approximately 60%, 20%, and 20% of the total sample size, respectively. Stratification was implemented when splitting training, validation, and test sets so that all had the same ratio of weak to healthy hand grip strength labels (approx. 16%/84% using a T-score of -2 and 46%/54% using a T-score of -1). Synthetic Minority Over-sampling Technique (SMOTE) was carried out on the training set. Additionally, all the features were standardized before feature selection.

The features were fed into a supervised-based classifier developed in Python 3 (Python Software Foundation, Delaware, US). The classifier considered in this analysis was a bagging ensemble model with additional balancing using XGBoost as the base estimator. Balanced accuracy was used as the metric to quantify the goodness-of-fit comparing the predictions of the classifier with the real labels. A grid search was employed on the training set to attain optimal values for the model hyper-parameters. Model fitting and feature selection (based on Select K Best using f_classification as a scoring function) were deployed simultaneously. For each combination of hyper-parameters’ values, a 5-fold cross-validation was carried out on the training data and the related balanced accuracy was obtained. The combination of hyper-parameters that returned the highest balanced accuracy was considered as the optimum and the selected model was evaluated on the validation set to prove its generalizability. Consecutively, training and validation sets were merged into a single new training set, the model was re-trained (with the optimal hyper-parameters and features selected), and the balanced accuracy was obtained for the test dataset. This procedure was carried out for the set of binary classification labels defined using a T-score of -2 and subsequently repeated for those defined using a T-score of -1.

## Results

### Regression task

Table 3 show the regression results obtained separately for males and females. As shown, the models built do not present any overfitting, since there is no statistically significant difference in performance across training, validation, and test sets. Moreover, the results obtained show an improvement (*p* < 0.001, for both male and female models) compared to the benchmark provided by a dummy regressor simply predicting the mean of the training data. Even though this improvement seems limited in absolute terms (< 0.5 Kg), the MAE is still within the 5-6.5 Kg range which is considered to be a sufficiently rough estimate of meaningful changes in grip strength [46].

Moreover, it is evident how the model’s performance is not variant with different subjects’ splits (Figures A.2-A.3, supplementary material) showing the model’s overall robustness.

While age and anthropometrics data (height, weight) were always selected by the models (Figures A.2-A.3, supplementary material), also physical activity-related measurements were often selected highlighting how these variables could be correlated with hand grip strength for both genders. Interestingly, the mean absolute error is substantially improved in females compared to males.

**Table 3:** Hand grip strength average and SD MAE across all iterations the most selected model (Linear Regression for males, Bayesian Ridge Regression for females)

Male				
	Training	Validation	Test	Benchmark
Average	6.29	6.32	6.32	6.59
SD	0.03	0.03	0.13	0.12
Range	0.16	0.16	0.63	0.58
Min	6.21	6.24	6.02	6.28
Max	6.37	6.4	6.65	6.86
Female				
Average	4.50	4.51	4.53	4.92
SD	0.02	0.02	0.08	0.1
Range	0.09	0.09	0.35	0.44
Min	4.46	4.47	4.34	4.71
Max	4.55	4.56	4.69	5.15

### Classification task

Tables 4 and 5 instead show the results achieved with the classification analysis. As expected, a T-score − 2 increases the data imbalance compared to a T-score of -1. Both models improve over the dummy classifier benchmark (*p*-value < 0.05), however the AUC-ROC in the first case is barely acceptable (0.70–0.76 for males and females respectively) while in the second case is performing well (0.86–0.87). ROC curves are shown in Figure A.4 in the supplementary material. Again, female subjects showed better performance in accordance with the regression models above. The results obtained indicate that, even if the absolute hand grip strength value is not predicted with an accuracy level sufficiently elevated from only accelerometry and demographic data, the characteristics that can be extrapolated from such variables (e.g., frail/pre-frail vs. healthy status) are detected in a viable way with acceptably accurate results. However, this depends on the T-score level considered for the definition of frailty/pre-frailty. While there is no general consensus on such cut-off values for the general population, some cut-offs have been proposed for particular cohorts and conditions. For instance, T-score < -1 SD is an indication of osteoporotic subjects [47], thus it could show how the developed models could potentially be adopted to detect osteoporotic subjects in the general population by simply fitting them with a wrist-worn device and limited information on age and anthropometrics.

It is interesting to note how those results are also generalizable in the analysed population, since results are not affected by the health conditions (cancer, diabetes, etc.) of the subjects.

**Table 4:** Training, validation and test balanced accuracy (stratified for the different health conditions) - classification results for labels defined using a T-score of -2 and − 1

Gender	T-score	Balanced Accuracy	Cancer (*n* = 61)	Diabetes (*n* = 88)	Cardiac Diseases (*n* = 244)	Respiratory Diseases (*n* = 230)	Musculoskeletal Diseases (*n* = 152)	Multimorbidity (*n* = 217)
Male	-2	Training	0.68	0.68	0.68	0.68	0.68	0.68
		Validation	0.62	0.62	0.62	0.62	0.62	0.62
		Test	0.71	0.51	0.63	0.53	0.57	0.65
		Benchmark	0.5	0.5	0.5	0.5	0.5	0.5
	-1	Training	0.8	0.8	0.8	0.8	0.8	0.8
		Validation	0.77	0.77	0.77	0.77	0.77	0.77
		Test	0.76	0.85	0.83	0.8	0.79	0.8
		Benchmark	0.5	0.5	0.5	0.5	0.5	0.5
Female	-2	Training	0.69	0.69	0.69	0.69	0.69	0.69
		Validation	0.67	0.67	0.67	0.67	0.67	0.67
		Test	0.59	0.60	0.7	0.69	0.71	0.67
		Benchmark	0.5	0.5	0.5	0.5	0.5	0.5
	-1	Training	0.8	0.8	0.8	0.8	0.8	0.8
		Validation	0.77	0.77	0.77	0.77	0.77	0.77
		Test	0.98	0.73	0.83	0.81	0.82	0.76
		Benchmark	0.5	0.5	0.5	0.5	0.5	0.5

*Results for the Neurodegenerative Diseases not reported as there were too few cases in the test set (*n* = 7)

## Discussion

In the last few years, the potential utility of wearable devices for senior citizens has gained significant research attention, with particular focus on wrist-worn activity trackers. The significance of commercial wearables offering clinical-grade insights is poised to escalate even further, given the demographic shift toward an aging population in Western societies. This demographic change accentuates the need for preventive medicine, self-health monitoring, remote patient care, and telemedicine, all of which can benefit from wearable technologies.

Hand grip strength has emerged as a compelling biomarker for aging, offering insights into various facets of health. This study’s dual objectives are to investigate the feasibility of using seven days of physical activity data, demographics, and questionnaire responses, coupled with ML models, to estimate hand grip strength in middle-aged and older adults, and to develop ML models capable of detecting specific medical conditions based on grip strength categorizations.

The regression analysis results (Table 3) reveal that the performance of the developed models is notably superior to a simple mean-predicting dummy regressor (*p*-value < 0.001). While the improvement in absolute terms may appear modest, the mean absolute error falls within the range considered sufficiently accurate for grip strength estimation [46].

The classification analysis (Tables 4 and 5), while affected by data imbalance, shows that the models outperform a benchmark dummy classifier (*p*-value < 0.05). Particularly, the models excel in categorizing individuals as frail/pre-frail, or healthy (AUC-ROC going from 0.70 to 0.76 to 0.86–0.87), depending on the T-score levels applied for frailty/pre-frailty definition. While cut-off values for frailty vary (Table 2), the results suggest that the models can viably detect characteristics associated with frailty.

**Table 5:** Additional performance metrics on test set (regardless of health conditions)- classification results for labels defined using a T-score of -2 and − 1

	Male		Female	
T-score	-2	-1	-2	-1
Accuracy	0.73	0.78	0.76	0.77
Balanced Accuracy	0.64	0.79	0.68	0.77
Sensitivity	0.76	0.73	0.81	0.76
Specificity	0.53	0.86	0.54	0.79
Geometric Mean Score	0.63	0.79	0.66	0.77
Recall Score	0.73	0.78	0.76	0.77
Precision Score	0.83	0.80	0.79	0.77
F1 Score	0.79	0.76	0.80	0.76
Area under the ROC Curve (AUC-ROC)	0.70	0.86	0.76	0.87

The analysis carried out in this investigation can be extended to other measures beside physical activity data collected in free-living settings. Given that frailty/pre-frailty are also correlated to other clinical measurements (i.e., gait speed [48]), it might be possible to use wearable devices, such as IMUs data, along with machine learning models to predict frailty/pre-frailty also through other activities easily implementable in free-living settings, such as stair climb [49], or the six minutes walking test [50].

In conclusion, this study presents a novel approach that harnesses consumer-grade wearables and machine learning to estimate hand grip strength and detect conditions like frailty. By leveraging everyday physical activity data, demographics, and ML techniques, the study contributes to the ongoing exploration of wearables as valuable tools for preventive medicine and remote patient monitoring. The results underscore the potential of wearables to provide clinically relevant insights, paving the way for more accessible and proactive healthcare practices. Further research in this domain holds promise for enhancing the well-being of older adults and expanding the horizons of wearable technology in healthcare.

## Conclusion

This study highlights the growing potential of wearables, especially wristbands, to provide clinically relevant data for senior citizens, driven by the aging population and the need for advanced healthcare solutions. Hand grip strength has emerged as a key biomarker of aging, and this research explores its association with free-living physical activity data via machine learning. The results show that our models outperform baseline approaches, offering promising accuracy in detecting conditions like frailty. In conclusion, this study underscores the potential of wearables and machine learning for proactive healthcare. Future research can further enhance older adults’ well-being and expand wearable technology’s role in healthcare.

### Electronic supplementary material

Below is the link to the electronic supplementary material.

**Supplementary Material 1:Appendix 1.** Participants categorization

**Supplementary Material 2: Appendix 2.**
Table A.1. Descriptions of terms used in variable names produced by GGIR.
Table A.2. Participant Features.

**Supplementary Material 3: Appendix 3.**
Figure A.1. Data distributions for both genders for hand grip strength, age, and BMI (*N* = 14,161).
Figure A.2. MAE and hand grip strength parameters selected over all iterations (male).
Figure A.3. MAE and hand grip strength parameters selected over all iterations (female).
Figure A.4. ROC curves for frailty classification of males using a T-score of -2 (top left), males using a T-score of -1 (top right), females using a T-score of -2 (bottom left), and females using a T-score of -1 (bottom right).

## Data Availability

This research has been conducted using the UK Biobank Resource under Application Number 47845.
