# Supplementary material for: Association between wrist-worn free-living accelerometry and hand grip strength in middle-aged and older adults
Source: Aging Clin Exp Res. 2024 May 8;36(1):108. doi: 10.1007/s40520-024-02757-z (PMC11078825; doi:10.1007/s40520-024-02757-z)
Supplement: Supplementary file 1 — Supplementary Material 1:Appendix 1. Participants categorization [file 40520_2024_2757_MOESM1_ESM.docx]

**Appendix 1.**

Participants Categorization.

- *Cancer*

Lung cancer

Breast cancer: History of breast cancer during verbal interview with trained nurse

Colorectal cancer: History of large bowel cancer/colorectal cancer, colon cancer/sigmoid cancer or rectal cancer during verbal interview with trained nurse

Skin cancer: History of skin cancer, malignant melanoma, non‐melanoma skin cancer, basal cell carcinoma or squamous cell carcinoma during verbal interview with trained nurse

Prostate cancer: History of prostate cancer during verbal interview with trained nurse

Cervical cancer: History of cervical cancer or cin cells at the cervix during verbal interview with trained nurse

Other cancer: History of any other cancer than lung cancer, breast cancer, colorectal cancer, skin cancer, prostate cancer or cervical malignancy during verbal interview with trained nurse

- *Diabetes*

Type 2 Diabetes: Inverse variance weighted fixed effects meta‐analysis of DIAGRAM Exome Consortium outcome (type 2 diabetes) and UK Biobank (history of diabetes unspecified, type 2 diabetes during verbal interview with trained nurse or current use of insulin medication)

- *Cardiac diseases*

Coronary heart disease

Inverse variance weighted fixed effects meta‐analysis of CARDIOGRAM Exome Consortium(4) outcome (coronary heart disease) and UK Biobank outcome:   (1) Myocardial infarction (MI), coronary artery bypass grafting, or coronary artery angioplasty documented in medical history at time of enrollment by a trained nurse or   (2) Hospitalization for ICD‐10 code for acute myocardial infarction (I21.0, I21.1, I21.2, I21.4, I21.9) or   (3) Hospitalization for OPCS‐4 coded procedure: coronary artery bypass grafting (K40.1‐40.4, K41.1‐41.4, K45.1‐45.5) or   (4) Hospitalization for OPCS‐4 coded procedure: coronary angioplasty ± stenting (K49.1‐49.2, K49.8‐49.9, K50.2, K75.1‐75.4, K75.8‐75.9)

Atrial fibrillation/flutter: History of atrial fibrillation or flutter during verbal interview with trained nurse or hospitalization for ICD code I48

Heart failure: History of heart failure during verbal interview with trained nurse or hospitalization for ICD code I50

Peripheral vascular disease: History of peripheral vascular disease or intermittent claudication during verbal interview with trained nurse or hospitalization for ICD code I74 or I1739

Aortic stenosis: History of aortic stenosis during verbal interview with trained nurse or hospitalization for ICD code I350

Venous thromboembolism: History of venous thromboembolism, deep vein thrombosis or pulmonary embolism during verbal interview with trained nurse or hospitalization for ICD code I26 or I80‐I82

Stroke: History of stroke, ischaemic stroke, or subarachnoid hemorrhage during verbal interview with trained nurse or hospitalization for ICD codes I60‐ I64

- *Chest diseases / respiratory*

Asthma: History of asthma during verbal interview with trained nurse

COPD/Emphysema: History of chronic obstructive airways disease, emphysema/chronic bronchitis or emphysema during verbal interview with trained nurse

Pneumonia: History of pneumonia during verbal interview with trained nurse

Hayfever: History of hayfever during verbal interview with trained nurse

- *Musculoskeletal conditions*

Back pain

Joint pain

Osteoporosis: History of osteoporosis during verbal interview with trained nurse

Osteoarthritis: History of osteoarthritis during verbal interview with trained nurse

Sciatica: History of sciatica during verbal interview with trained nurse

Prolapsed disc: History of prolapsed disc/slipped disc during verbal interview with trained nurse

- *Neurodegenerative diseases*

Alzheimer’s disease (AD) and other dementias

Parkinson’s disease (PD) and PD-related disorders

Prion disease

Motor neurone diseases (MND)

Huntington’s disease (HD)

Spinocerebellar ataxia (SCA)

Spinal muscular atrophy (SMA)
