# Supplementary material for: Association between wrist-worn free-living accelerometry and hand grip strength in middle-aged and older adults
Source: Aging Clin Exp Res. 2024 May 8;36(1):108. doi: 10.1007/s40520-024-02757-z (PMC11078825; doi:10.1007/s40520-024-02757-z)
Supplement: Supplementary file 2 — Supplementary Material 2: Appendix 2. Table A.1. Descriptions of terms used in variable names produced by GGIR. Table A.2. Participant Features. [file 40520_2024_2757_MOESM2_ESM.docx]

**Appendix 2.**

Table A.1. Descriptions of terms used in variable names produced by GGIR.

### Person level summary

| **(Part of) variable name** | **Description** |
| --- | --- |
| ID | Participant id |
| device_sn | Device serial number |
| bodylocation | Body location extracted from file header |
| filename | Name of the data file |
| start_time | Timestamp when recording started |
| startday | Day of the week on which recording started |
| samplefreq | Sample frequency (Hz) |
| device | Accelerometer brand, e.g. GENEACtiv |
| clipping_score | The Clipping score: Fraction of 15 minute windows per file for which the acceleration in one of the three axis was close to the maximum for at least 80% of the time. This should be 0. |
| meas_dur_dys} | Measurement duration (days) |
| complete_24hcycle | Completeness score: Fraction of 15 minute windows per 24 hours for which no valid data is available at any day of the measurement. |
| meas_dur_def_proto_day | measurement duration according to protocol (days): Measurement duration (days) minus the hours that are ignored at the beginning and end of the measurement motived by protocol design |
| wear_dur_def_proto_day | wear duration duration according to protocol (days): So, if the protocol was seven days of measurement then wearing the accelerometer for 8 days and recording data for 8 days will still makethat the wear duration is 7 days |
| calib_err | Calibration error (static estimate) Estimated based on all ‘non-movement’ periods in the measurement after applying the autocalibration. |
| calib_status | Calibration status: Summary statement about the status of the calibration error minimisation |
| ENMO_fullRecordingMean | ENMO is the main summary measure of acceleration. The value presented is the average ENMO over all the available data normalised per 24 hour cycles (diurnal balanced), with invalid data imputed by the average at similar time points on different days of the week. In addition to ENMO it is possible to extract other acceleration metrics (i.e. BFEN, HFEN, HFENplus). We emphasize that it is calculated over the full recording because the alternative is that a variable is only calculated overmeasurement days with sufficient valid hours of data. |
| ENMO | (only available if set to true in part1.R) ENMO is the main summary measure of acceleration. The value presented is the average ENMO over all the available data normalised per 24 hour cycles, with invalid data imputed by the average at similar timepoints on different days of the week. In addition to ENMO it is possible to extract other acceleration metrics in part1.R (i.e. BFEN, HFEN, HFENplus) See also [van Hees PLoSONE April 2013](http://journals.plos.org/plosone/article?id=10.1371/journal.pone.0061691) for a detailed description and comparison of these techniques. |
| pX_A_mg_0-24h_fullRecording | This variable represents the Xth percentile in the distribution of short epoch metric value A of the average day. The average day may not be ideal for describing the distirbution. Therefore, the code also extracts the following variable. |
| AD_pX_A_mg_0-24h | This variable represents the Xth percentile in the distribution of short epoch metric value A per day averaged across all days. |
| L5_A_mg_0-24 | Average of metric A during the least active five* hours in the day that is the lowest rolling average value of metric A. (* window size is modifiable by argument winhr) |
| M5_A_mg_0-24 | Average of metric A during the most active five* hours in the day that is the lowest rolling average value of metric A. (* window size is modifiable by argument winhr) |
| L5hr_A_mg_0-24 | Starting time in hours and fractions of hours of L5_A_mg_0-24 |
| M5hr_A_mg_0-24 | Starting time in hours and fractions of hours of M5_A_mg_0-24 |
| ig_gradient_ENMO_0-24hr_fullRecording | Intensity gradient calculated over the full recording. |
| 1to6am_ENMO_mg | Average metric value ENMO between 1am and 6am |
| N valid WEdays | Number of valid weekend days |
| N valid WKdays | Number of valid week days |
| IS_interdailystability | inter daily stability |
| IV_intradailyvariability | intra daily variability |
| IVIS_windowsize_minutes | Sizes of the windows based on which IV and IS are calculated (note that this is modifiable) |
| IVIS_epochsize_seconds | size of the epochs based on which IV and IS are calculated (note that this is modifiable) |
| AD_ | All days (plain average of all available days, no weighting). The variable was calculated per day and then averaged over all the available days |
| WE_ | Weekend days (plain average of all available days, no weighting). The variable was calculated per day and then averaged over weekend days only |
| WD_ | Week days (plain average of all available days, no weighting). The variable was calculated per day and then averaged over week days only |
| WWE_ | Weekend days (weighted average) The variable was calculated per day and then averaged over weekend days. Double weekend days are averaged. This is only relevant for experiments that last for more than seven days. |
| WWD_ | Week days (weighted average) The variable was calculated per day and then averaged over week days. Double weekend days were averaged. This is only relevant for experiments that last for more than seven days) |
| WWD_MVPA_E5S_T100_ENMO | Time spent in moderate-to-vigorous based on 5 second epoch size and an ENMO metric threshold of 100 |
| WWE_MVPA_E5S_B1M80%_T100_ENMO | Time spent in moderate-to-vigorous based on 5 second epoch size and an ENMO metric threshold of 100 based on a bout criteria of 100 |
| WE_[100,150)_mg_0-24h_ENMO | Time spent between (and including) 100 mg and 150 (excluding 150 itself) between 0 and 24 hours (the full day) using metric ENMO data exclusion strategy (value=1, ignore specific hours; value=2, ignore all data before the first midnight and after the last midnight) |
| _MVPA_E5S_B1M80_T100 | MVPA calculated based on 5 second epoch setting bout duration 1 Minute and inclusion criterion of more than 80 percent. This is only done for metric ENMO at the moment, and only if mvpathreshold is not left blank |
| _ENMO_mg | ENMO or other metric was first calcualte per day and then average according to AD, WD, WWE, WWD |
| data exclusion strategy | A log of the decision made when calling g.impute: value=1 mean ignore specific hours; value=2 mean ignore all data before the first midnight and after the last midnight |
| n hours ignored at start of meas (if strategy=1) | number of hours ignored at the start of the measurement (if strategy = 1) A log of decision made in part2.R |
| n hours ignored at end of meas (if strategy=1) | number of hours ignored at the end of the measurement (if strategy = 1). A log of decision made in part2.R |
| n hours ignored at end of meas (if strategy=1) | number of days of measurement after which all data is ignored (if strategy = 1) A log of decision made in part2.R |
| epoch size to which acceleration was averaged (seconds) | A log of decision made in part1.R |
| pdffilenumb | Indicator of in which pdf-file the plot was stored |
| pdfpagecount | Indicator of in which pdf-page the plot was stored |

### Day level summary

| **(Part of) variable name** | **Description** |
| --- | --- |
| ID | Participant id |
| filename | Name of the data file |
| calender_date | Timestamp and date on which measurement started |
| bodylocation | Location of the accelerometer as extracted from file header |
| N valid hours | Number of hours with valid data in the day |
| N hours | Number of hours of measurement in a day, which typically is 24, unless it is a day on which the clock changes (DST) resulting in 23 or 25 hours. The value can be less than 23 if the measurement started or ended this day |
| weekday | Name of weekday |
| measurement | Day of measurement Day number relative to start of the measurement |
| L5hr_ENMO_mg_0-24h | Hour on which L5 starts for these 24 hours (defined with metric ENMO) |
| L5_ENMO_mg_0-24h | Average acceleration for L5 (defined with metric ENMO) |
| [A,B)_mg_0-24h_ENMO | Time spent in minutes between (and including) acceleration value A in mg and (excluding) acceleration value B in mg based on metric ENMO |
| ig_gradient_ENMO_0-24hr | Gradient from intensity gradient analysis (Rowlands et al 2018) based on metric ENMO for the time segment 0 to 24 hours |
| ig_intercept_ENMO_0-24hr | Intercept from intensity gradient analysis (Rowlands et al 2018) based on metric ENMO for the time segment 0 to 24 hours |
| ig_rsquared_ENMO_0-24hr | r squared from intensity gradient analysis (Rowlands et al 2018) based on metric ENMO for the time segment 0 to 24 hours |

### Night level summaries

| **(Part of) variable name** | **Description** |
| --- | --- |
| ID | Participant ID extracted from file |
| night | Number of the night in the recording |
| sleeponset | Detected onset of sleep expressed as hours since the midnight of the previous night. |
| wakeup | Detected waking time (after sleep period) expressed as hours since the midnight of the previous night. |
| SptDuration | Difference between onset and waking time. |
| sleepparam | Definition of sustained inactivity by accelerometer. |
| guider | guider used (see paragraph ‘Waking-waking or 24 hour time-use analaysis’). |
| guider_onset | Start of Sleep Period Time window derived from the guider. |
| guider_wake | End of Sleep Period Time window derived guider. |
| guider_SptDuration | Time SPT duration derived from guider_wake and guider_onset. |
| error_onset | Difference between sleeponset and guider_onset |
| error_wake | Difference between wakeup and guider_wake |
| fraction_night_invalid | Fraction of the night (noon-noon or 6pm-6pm) for which the data was invalid, e.g. monitor not worn or no accelerometer measurement started/ended within the night. |
| SleepDurationInSpt | Total sleep duration, which equals the accumulated nocturnal sustained inactivity bouts within the Sleep Period Time. |
| duration_sib_wakinghours | Accumulated sustained inactivty bouts during the day. These are the periods we would label during the night as sleep, but during the day they form a subclass of inactivity, which may represent day time sleep or wakefulness while being motionless for a sustained period of time number_sib_sleepperiod} Number of noturnal sleep periods, with nocturnal referring to the Sleep Period Time window. |
| duration_sib_wakinghours_atleast15min | Same as duration_sib_wakinghours, but limited to SIBs that last at least 15 minutes. |
| number_sib_wakinghours | Number of sustained inactivity bouts during the day, with day referring to the time outside the Sleep Period Time window. |
| sleeponset_ts | sleeponset formatted as a timestamp |
| wakeup_ts | wakeup formatted as a timestamp |
| guider_onset_ts | guider_onset formatted as a timestamp |
| guider_wake_ts | guider_wake formatted as a timestamp |
| page | pdf page on which the visualisation can be found |
| daysleeper | If 0 then the person is a nightsleeper (sleep period did not overlap with noon) if value=1 then the person is a daysleeper (sleep period did overlap with noon) |
| weekday | Day of the week on which the night started |
| calendardate | Calendar date on which the night started |
| filename | Name of the accelerometer file |
| cleaningcode | see paragraph ‘Waking-waking or 24 hour time-use analaysis’). |
| sleeplog_used | Whether a sleep log was used (TRUE/FALSE) |
| acc_available | Whether accelerometer data was available (TRUE/FALSE). |

### Person level summaries

| **(Part of) variable name** | **Description** |
| --- | --- |
| _mn | mean across days |
| _sd | standard deviation across days |
| _AD | All days |
| _WE | Weekend days |
| _WD | Week days |
| sleeplog_used | Whether a sleeplog was available (TRUE) or not (FALSE) |
| sleep_efficiency | Accelerometer detrive sleep efficiency within the sleep period time calculatd as the ratio between acc_SleepDurationInSpt and acc_SptDuration (denominator). Only available at person level, because at night level the user can calculate this from existing variables. |
| n_nights_acc | Number of nights of accelerometer data |
| n_nights_sleeplog | Number of nights of sleeplog data. |
| n_WE_nights_complete | Number of weekend nights complete which means both accelerometer and estimate from guider. |
| n_WD_nights_complete | Number of weekday nights complete which means both accelerometer and estimate from guider. |
| n_WEnights_daysleeper | Number of weekend nights on which the person slept until after noon. |
| n_WDnights_daysleeper | Number of weekday nights on which the person slept until after noon. |
| duration_sib_wakinghour | Total duration of sustained inactivity bouts during the waking hours. |
| number_sib_wakinghours | Number of sustained inactivity bouts during the waking hours. |
| average_dur_sib_wakinghours | Average duration of the sustained inactivity bouts during the day (outside the sleep period duration). Calculated as duration_sib_wakinghour divided by number_sib_wakinghours per day, after which the mean and standard deviation are calculated across days. |

### Day level summary

| **(Term in) variable name** | **Description** |
| --- | --- |
| sleeponset | onset of sleep expressed in hours since the midnight in the night preceding the night of interest, e.g. 26 is 2am. |
| wakeup | waking up time express in the same way as sleeponset. |
| sleeponset_ts | onset of sleep expressed as a timestamp hours:minutes:seconds |
| daysleeper | if 0 then the person woke up before noon, if 1 then the person woke up after noon |
| cleaningcode | See paragraph [Sleep and full day time-use analysis in GGIR](https://cran.r-project.org/web/packages/GGIR/vignettes/GGIR.html#Sleep%20and%20full%20day%20time-use%20analysis%20in%20GGIR). |
| dur_day_spt_min | Total length of daytime waking hours and spt combined (typically 24 hours for MM report). |
| dur_ | duration of a behavioral class that will be specified int he rest of the variable name |
| ACC_ | (average) acceleration according to default metric specific by acc.metric |
| _spt_wake_ | Wakefulness within the Sleep period time window. |
| _spt_sleep_ | Sleep within the Sleep period time window. |
| _IN_ | Inactivity |
| _LIG_ | Light activity |
| _MOD_ | Moderate activity |
| _VIG_ | Vigorous activity |
| _MVPA_ | Moderate or Vigorous activity |
| _unbt_ | Unbouted |
| _bts_ | Bouts (also known as sojourns), which are segments that for which the acceleration is within a specified range for a specified fraction of the time. |
| _bts_1_10_ | Bouts lasting at least 1 minute and less than 10 minutes (1 and 9.99 minutes are included, but 10 minutes is not). |
| Nblock | number of blocks of a certain behavioral class, not these are not bouts but a count of the number of times the behavioral class occures without interruptions. |
| WW | in filename refers to analyses based on the timewindow from waking to waking up |
| MM | in filename refers to analyses done on windows between midnight and midnight |
| _total_IN | total time spent in inactivity (no distinction between bouted or unbouted behavior, this is a simple count of the number of epochs that meet the threshold criteria. |
| _total_LIG | total time spent in light activity. |
| nonwear_perc_day_spt | Non-wear percentage during the waking hours of this day. |
| nonwear_perc_spt | Non-wear percentage during the spt hours of this day. |
| nonwear_perc_day | Non-wear percentage during the whole day, including waking and spt. |
| dur_day_min | Duration of waking hours within this day window |
| dur_spt_min | Duration of Sleep Period Time wihtin this day window. |
| dur_day_spt_min | Duration this day window, including both waking hours and SPT. |
| sleep_efficiency | Sleep efficiency (within the SPT window, not including sleep latency) |
| L5TIME | Timing of least active 5hrs |
| M5TIME | Timing of most active 5hrs |
| L5VALUE | Acceleration value for least active 5hrs |
| M5VALUE | Acceleration value for most active 5hrs |

### Person level summary

Most variables in the person level summary are derived from the day level summary, but extended with _pla to indicate that the variable was calculated as the plain average across all valid days. Variables extended with _wei represent the weighted average of across all days where weekend days always weighted 2/5 relative to the contribution of week days.

| **Variable name** | **Description** |
| --- | --- |
| Nvaliddays | Total number of valid days. |
| Nvaliddays_WD | Number of valid week days. |
| Nvaliddays_WE | Number of valid weekend days, where the days that start on Saturday or Sunday are considered weekend. |
| NcleaningcodeX | Number of days that had cleaning code X for the corresponding sleep analysis in part 4. In case of MM analysis this refers to the night at the end of the day. |

Table A.2. Participant Features.

Feature Name Description

| Hand Grip Strength | right and left hand isometric grip strength, measured using a Jamar J00105 hydraulic hand dynamometer (kg) |
| --- | --- |
| Sex | 0-female 1-male |
| Age | years |
| Ethnicity | 1-White 1001-British 2001-White and Black Caribbean 3001-Indian 4001-Caribbean 2-Mixed 1002 Irish 2002-White and Black African 3002-Pakistani 4002-African 3-Asian or Asian British 1003-Any other white background 2003-White and Asian 3003-Bangladeshi 4003-Any other Black background 4-Black or Black British 2004-Any other mixed background 3004-Any other Asian background 5-Chinese 6-Other ethnic group -1-Do not know -3-Prefer not to answer |
| BMI | body mass index |
| Weight | (kg) |
| Estimated Height | sqrt(weight/BMI) (m) |
| Cardiac Diseases | 0-none 1-diagnosed |
| Neurodegenerative Diseases | 0-none 1-diagnosed |
| Respiratory Diseases | 0-none 1-diagnosed |
| Musculoskeletal Diseases | 0-none 1-diagnosed |
| Multimorbidity | 0-none 1-diagnosed |
| summary.clipping_score | A summary measure indicating the fraction of 15-minute windows per file for which acceleration in one of the three axes was close to the maximum for at least 80% of the time. This score should ideally be 0. |
| summary.meas_dur_dys | Summary measure representing the measurement duration in days. |
| summary.complete_24hcycle | Summary measure indicating the fraction of 15-minute windows per 24 hours for which no valid data is available at any day of the measurement. |
| summary.meas_dur_def_proto_day | Summary measure representing the measurement duration according to protocol (days), which is the measurement duration minus the hours ignored at the beginning and end of the measurement due to protocol design. |
| summary.wear_dur_def_proto_day | Summary measure representing the wear duration duration according to protocol (days), which takes into account the protocol design when calculating the wear duration. |
| summary.calib_err | Summary measure representing the calibration error (static estimate) calculated based on non-movement periods in the measurement after applying autocalibration. |
| summary.calib_status | Summary statement indicating the status of the calibration error minimization process. |
| summary.ENMO_fullRecordingMean | Summary measure of ENMO (main summary measure of acceleration) normalized per 24-hour cycles over all available data, imputing invalid data with averages at similar time points on different days of the week. |
| summary.N.valid.WEdays | Summary measure indicating the number of valid weekend days. |
| summary.N.valid.WKdays | Summary measure indicating the number of valid weekdays. |
| summary.IS_interdailystability | Summary measure of inter-daily stability. |
| summary.IV_intradailyvariability | Summary measure of intra-daily variability. |
| summary.IVIS_windowsize_minutes | Summary measure indicating the size of windows in minutes based on which IV and IS are calculated. |
| summary.IVIS_epochsize_seconds | Summary measure indicating the size of epochs in seconds based on which IV and IS are calculated. |
| summary.AD_L5hr_ENMO_mg_0.24hr | Summary measure representing the average acceleration for the least active five hours in the day using metric ENMO over the available data. |
| summary.WE_L5hr_ENMO_mg_0.24hr | Summary measure representing the average acceleration for the least active five hours in the weekend days using metric ENMO over the available data. |
| summary.WD_L5hr_ENMO_mg_0.24hr | Summary measure representing the average acceleration for the least active five hours in the week days using metric ENMO over the available data. |
| summary.WWE_L5hr_ENMO_mg_0.24hr | Summary measure representing the average acceleration for the least active five hours in the weighted average of weekend days using metric ENMO over the available data. |
| summary.WWD_L5hr_ENMO_mg_0.24hr | Summary measure representing the average acceleration for the least active five hours in the weighted average of week days using metric ENMO over the available data. |
| summary.AD_L5hr_ENMO_mg_0.24hr.1 | Summary measure representing the average acceleration for the least active five hours in the day using metric ENMO over the available data (a duplicated variable name). |
| summary.WE_L5hr_ENMO_mg_0.24hr.1 | Summary measure representing the average acceleration for the least active five hours in the day using metric ENMO over the available data (a duplicated variable name). |
| summary.AD_L5_ENMO_mg_0.24hr | Summary of the average ENMO (a measure of acceleration) during the least active 5 hours within the day for all days. |
| summary.AD_M5_ENMO_mg_0.24hr | Summary of the average ENMO during the most active 5 hours within the day for all days. |
| summary.AD_M5hr_ENMO_mg_0.24hr | Summary of the starting time (in hours) of the most active 5-hour period for all days. |
| summary.AD_mean_ENMO_mg_0.24hr | Summary of the mean of ENMO values throughout the day for all days. |
| summary.AD_mean_ENMO_mg_1.6am | Summary of the mean ENMO value between 1:00 AM and 6:00 AM for all days. |
| summary.AD_MVPA_E1M_T100_ENMO_0.24hr | Summary of time spent in moderate-to-vigorous physical activity (MVPA) based on a 1-minute epoch size and a threshold of 100 in the ENMO metric for all days. |
| summary.AD_MVPA_E5M_T100_ENMO_0.24hr | Summary of time spent in MVPA with a 5-minute epoch size and a threshold of 100 in the ENMO metric for all days. |
| summary.AD_MVPA_E5S_B10M80._T100_ENMO_0.24hr | Summary of time spent in MVPA with a 5-second epoch size, an ENMO threshold of 100, and a minimum bout duration of 10 minutes for all days. |
| summary.AD_MVPA_E5S_B1M80._T100_ENMO_0.24hr | Summary of time spent in MVPA with a 5-second epoch size, an ENMO threshold of 100, and a minimum bout duration of 1 minute for all days. |
| summary.AD_MVPA_E5S_B5M80._T100_ENMO_0.24hr | Summary of time spent in MVPA with a 5-second epoch size, an ENMO threshold of 100, and a minimum bout duration of 5 minutes for all days. |
| summary.AD_MVPA_E5S_T100_ENMO_0.24hr | Summary of time spent in MVPA with a 5-second epoch size and an ENMO threshold of 100 for all days. |
| summary.data.exclusion.stategy. .value.1. .ignore.specific.hours. .value.2. .ignore.all.data.before.the.first.midnight.and.after.the.last.midnight. | Summary of the data exclusion strategy used: value 1 indicates ignoring specific hours, value 2 indicates ignoring data before the first midnight and after the last midnight. |
| summary.WD_L5_ENMO_mg_0.24hr | Summary of the average ENMO during the least active 5 hours within the day for week days. |
| summary.WD_L5hr_ENMO_mg_0.24hr.1 | Summary of the starting time (in hours) of the least active 5-hour period for week days. |
| summary.WD_M5_ENMO_mg_0.24hr | Summary of the average ENMO during the most active 5 hours within the day for week days. |
| summary.WD_M5hr_ENMO_mg_0.24hr | Summary of the starting time (in hours) of the most active 5-hour period for week days. |
| summary.WD_mean_ENMO_mg_0.24hr | Summary of the mean of ENMO values throughout the day for week days. |
| summary.WD_mean_ENMO_mg_1.6am | Summary of the mean ENMO value between 1:00 AM and 6:00 AM for week days. |
| summary.WD_MVPA_E1M_T100_ENMO_0.24hr | Summary of time spent in MVPA based on a 1-minute epoch size and a threshold of 100 in the ENMO metric for week days. |
| summary.WD_MVPA_E5M_T100_ENMO_0.24hr | Summary of time spent in MVPA with a 5-minute epoch size and a threshold of 100 in the ENMO metric for week days. |
| summary.WD_MVPA_E5S_B10M80._T100_ENMO_0.24hr | Summary of time spent in moderate-to-vigorous physical activity (MVPA) based on a 5-second epoch size, an ENMO threshold of 100, and a minimum bout duration of 10 minutes for week days. |
| summary.WD_MVPA_E5S_B1M80._T100_ENMO_0.24hr | Summary of time spent in MVPA based on a 5-second epoch size, an ENMO threshold of 100, and a minimum bout duration of 1 minute for week days. |
| summary.WD_MVPA_E5S_B5M80._T100_ENMO_0.24hr | Summary of time spent in MVPA based on a 5-second epoch size, an ENMO threshold of 100, and a minimum bout duration of 5 minutes for week days. |
| summary.WD_MVPA_E5S_T100_ENMO_0.24hr | Summary of time spent in MVPA based on a 5-second epoch size and an ENMO threshold of 100 for week days. |
| summary.WE_L5_ENMO_mg_0.24hr | Summary of the average ENMO during the least active 5 hours within the day for weekend days. |
| summary.WE_M5_ENMO_mg_0.24hr | Summary of the average ENMO during the most active 5 hours within the day for weekend days. |
| summary.WE_M5hr_ENMO_mg_0.24hr | Summary of the starting time (in hours) of the most active 5-hour period for weekend days. |
| summary.WE_mean_ENMO_mg_0.24hr | Summary of the mean of ENMO values throughout the day for weekend days. |
| summary.WE_mean_ENMO_mg_1.6am | Summary of the mean ENMO value between 1:00 AM and 6:00 AM for weekend days. |
| summary.WE_MVPA_E1M_T100_ENMO_0.24hr | Summary of time spent in MVPA based on a 1-minute epoch size and a threshold of 100 in the ENMO metric for weekend days. |
| summary.WE_MVPA_E5M_T100_ENMO_0.24hr | Summary of time spent in MVPA with a 5-minute epoch size and a threshold of 100 in the ENMO metric for weekend days. |
| summary.WE_MVPA_E5S_B10M80._T100_ENMO_0.24hr | Summary of time spent in MVPA with a 5-second epoch size, an ENMO threshold of 100, and a minimum bout duration of 10 minutes for weekend days. |
| summary.WE_MVPA_E5S_B1M80._T100_ENMO_0.24hr | Summary of time spent in MVPA with a 5-second epoch size, an ENMO threshold of 100, and a minimum bout duration of 1 minute for weekend days. |
| summary.WE_MVPA_E5S_B5M80._T100_ENMO_0.24hr | Summary of time spent in MVPA with a 5-second epoch size, an ENMO threshold of 100, and a minimum bout duration of 5 minutes for weekend days. |
| summary.WE_MVPA_E5S_T100_ENMO_0.24hr | Summary of time spent in MVPA with a 5-second epoch size and an ENMO threshold of 100 for weekend days. |
| summary.WWD_L5_ENMO_mg_0.24hr | Summary of the average ENMO during the least active 5 hours within the day for weighted week days. |
| summary.WWD_L5hr_ENMO_mg_0.24hr.1 | Summary of the starting time (in hours) of the least active 5-hour period for weighted week days. |
| summary.WWD_M5_ENMO_mg_0.24hr | Summary of the average ENMO during the most active 5 hours within the day for weighted week days. |
| summary.WWD_M5hr_ENMO_mg_0.24hr | Summary of the starting time (in hours) of the most active 5-hour period for weighted week days. |
| summary.WWD_mean_ENMO_mg_0.24hr | Summary of the mean of ENMO values throughout the day for weighted week days. |
| summary.WWD_mean_ENMO_mg_1.6am | Mean ENMO (average acceleration) during week days (WWD) between 1am and 6am. |
| summary.WWD_MVPA_E1M_T100_ENMO_0.24hr | Time spent in moderate-to-vigorous physical activity (MVPA) based on 1 minute epoch size and ENMO metric threshold of 100 during week days (WWD), averaged over 24 hours. |
| summary.WWD_MVPA_E5M_T100_ENMO_0.24hr | Time spent in MVPA based on 5 minute epoch size and ENMO metric threshold of 100 during week days (WWD), averaged over 24 hours. |
| summary.WWD_MVPA_E5S_B10M80._T100_ENMO_0.24hr | Time spent in MVPA based on 5 second epoch size, bout duration of at least 10 minutes, and ENMO metric threshold of 100 during week days (WWD), averaged over 24 hours. |
| summary.WWD_MVPA_E5S_B1M80._T100_ENMO_0.24hr | Time spent in MVPA based on 5 second epoch size, bout duration of at least 1 minute, and ENMO metric threshold of 100 during week days (WWD), averaged over 24 hours. |
| summary.WWD_MVPA_E5S_B5M80._T100_ENMO_0.24hr | Time spent in MVPA based on 5 second epoch size, bout duration of at least 5 minutes, and ENMO metric threshold of 100 during week days (WWD), averaged over 24 hours. |
| summary.WWD_MVPA_E5S_T100_ENMO_0.24hr | Time spent in MVPA based on 5 second epoch size and ENMO metric threshold of 100 during week days (WWD), averaged over 24 hours. |
| summary.WWE_L5_ENMO_mg_0.24hr | Average acceleration (ENMO) during the least active 5 hours of the day for weekend days (WWE), averaged over 24 hours. |
| summary.WWE_L5hr_ENMO_mg_0.24hr.1 | Starting time in hours and fractions of hours of the least active 5 hours for weekend days (WWE), averaged over 24 hours. |
| summary.WWE_M5_ENMO_mg_0.24hr | Average acceleration (ENMO) during the most active 5 hours of the day for weekend days (WWE), averaged over 24 hours. |
| summary.WWE_M5hr_ENMO_mg_0.24hr | Starting time in hours and fractions of hours of the most active 5 hours for weekend days (WWE), averaged over 24 hours. |
| summary.WWE_mean_ENMO_mg_0.24hr | Mean ENMO (average acceleration) for weekend days (WWE), averaged over 24 hours. |
| summary.WWE_mean_ENMO_mg_1.6am | Mean ENMO (average acceleration) for weekend days (WWE) between 1am and 6am. |
| summary.WWE_MVPA_E1M_T100_ENMO_0.24hr | Time spent in MVPA based on 1 minute epoch size and ENMO metric threshold of 100 for weekend days (WWE), averaged over 24 hours. |
| summary.WWE_MVPA_E5M_T100_ENMO_0.24hr | Time spent in MVPA based on 5 minute epoch size and ENMO metric threshold of 100 for weekend days (WWE), averaged over 24 hours. |
| summary.WWE_MVPA_E5S_B10M80._T100_ENMO_0.24hr | Time spent in MVPA based on 5 second epoch size, bout duration of at least 10 minutes, and ENMO metric threshold of 100 for weekend days (WWE), averaged over 24 hours. |
| summary.WWE_MVPA_E5S_B1M80._T100_ENMO_0.24hr | Time spent in MVPA based on 5 second epoch size, bout duration of at least 1 minute, and ENMO metric threshold of 100 for weekend days (WWE), averaged over 24 hours. |
| summary.WWE_MVPA_E5S_B5M80._T100_ENMO_0.24hr | Time spent in MVPA based on 5 second epoch size, bout duration of at least 5 minutes, and ENMO metric threshold of 100 for weekend days (WWE), averaged over 24 hours. |
| summary.WWE_MVPA_E5S_T100_ENMO_0.24hr | Time spent in MVPA based on 5 second epoch size and ENMO metric threshold of 100 for weekend days (WWE), averaged over 24 hours. |
| SptDuration_AD_T5A5_mn | Average duration of the Sleep Period Time window during all days (AD) with a threshold of 5 to 5 hours. |
| SptDuration_AD_T5A5_sd | Standard deviation of the duration of the Sleep Period Time window during all days (AD) with a threshold of 5 to 5 hours. |
| SleepDurationInSpt_AD_T5A5_mn | Average sleep duration within the Sleep Period Time window during all days (AD) with a threshold of 5 to 5 hours. |
| SleepDurationInSpt_AD_T5A5_sd | Standard deviation of sleep duration within the Sleep Period Time window during all days (AD) with a threshold of 5 to 5 hours. |
| sleep_efficiency_AD_T5A5_mn | Sleep efficiency (ratio of sleep duration to Sleep Period Time) during all days (AD) with a threshold of 5 to 5 hours, averaged. |
| sleep_efficiency_AD_T5A5_sd | Standard deviation of sleep efficiency during all days (AD) with a threshold of 5 to 5 hours. |
| duration_sib_wakinghours_AD_T5A5_mn | Average duration of sustained inactivity bouts during waking hours during all days (AD) with a threshold of 5 to 5 hours. |
| duration_sib_wakinghours_AD_T5A5_sd | Standard deviation of the average duration of sustained inactivity bouts during waking hours during all days (AD) with a threshold of 5 to 5 hours. |
| number_sib_sleepperiod_AD_T5A5_mn | Average number of sustained inactivity bouts during the sleep period time window on all days (AD) with a threshold of 5 minutes for both sleep and wakefulness (T5A5). |
| number_sib_sleepperiod_AD_T5A5_sd | Standard deviation of the number of sustained inactivity bouts during the sleep period time window on all days (AD) with a threshold of 5 minutes for both sleep and wakefulness (T5A5). |
| number_sib_wakinghours_AD_T5A5_mn | Average number of sustained inactivity bouts during the waking hours on all days (AD) with a threshold of 5 minutes for both sleep and wakefulness (T5A5). |
| number_sib_wakinghours_AD_T5A5_sd | Standard deviation of the number of sustained inactivity bouts during the waking hours on all days (AD) with a threshold of 5 minutes for both sleep and wakefulness (T5A5). |
| duration_sib_wakinghours_atleast15min_AD_T5A5_mn | Average duration of sustained inactivity bouts during the waking hours of at least 15 minutes on all days (AD) with a threshold of 5 minutes for both sleep and wakefulness (T5A5). |
| duration_sib_wakinghours_atleast15min_AD_T5A5_sd | Standard deviation of the duration of sustained inactivity bouts during the waking hours of at least 15 minutes on all days (AD) with a threshold of 5 minutes for both sleep and wakefulness (T5A5). |
| average_dur_sib_wakinghours_AD_T5A5_mn | Average duration of sustained inactivity bouts during the waking hours on all days (AD) with a threshold of 5 minutes for both sleep and wakefulness (T5A5). |
| average_dur_sib_wakinghours_AD_T5A5_sd | Standard deviation of the average duration of sustained inactivity bouts during the waking hours on all days (AD) with a threshold of 5 minutes for both sleep and wakefulness (T5A5). |
| n_days_w_sib_wakinghours_AD_T5A5 | Number of days with sustained inactivity bouts during the waking hours on all days (AD) with a threshold of 5 minutes for both sleep and wakefulness (T5A5). |
| sleeponset_AD_T5A5_mn | Average sleep onset time expressed in hours since the midnight of the previous night on all days (AD) with a threshold of 5 minutes for both sleep and wakefulness (T5A5). |
| sleeponset_AD_T5A5_sd | Standard deviation of sleep onset time expressed in hours since the midnight of the previous night on all days (AD) with a threshold of 5 minutes for both sleep and wakefulness (T5A5). |
| wakeup_AD_T5A5_mn | Average waking up time expressed in hours since the midnight of the previous night on all days (AD) with a threshold of 5 minutes for both sleep and wakefulness (T5A5). |
| wakeup_AD_T5A5_sd | Standard deviation of waking up time expressed in hours since the midnight of the previous night on all days (AD) with a threshold of 5 minutes for both sleep and wakefulness (T5A5). |
| SptDuration_WD_T5A5_mn | Average Sleep Period Time duration on week days (WD) with a threshold of 5 minutes for both sleep and wakefulness (T5A5). |
| SptDuration_WD_T5A5_sd | Standard deviation of Sleep Period Time duration on week days (WD) with a threshold of 5 minutes for both sleep and wakefulness (T5A5). |
| SleepDurationInSpt_WD_T5A5_mn | Average sleep duration within the Sleep Period Time on week days (WD) with a threshold of 5 minutes for both sleep and wakefulness (T5A5). |
| SleepDurationInSpt_WD_T5A5_sd | Standard deviation of sleep duration within the Sleep Period Time on week days (WD) with a threshold of 5 minutes for both sleep and wakefulness (T5A5). |
| sleep_efficiency_WD_T5A5_mn | Average sleep efficiency within the Sleep Period Time on week days (WD) with a threshold of 5 minutes for both sleep and wakefulness (T5A5). |
| sleep_efficiency_WD_T5A5_sd | Standard deviation of sleep efficiency within the Sleep Period Time on week days (WD) with a threshold of 5 minutes for both sleep and wakefulness (T5A5). |
| duration_sib_wakinghours_WD_T5A5_mn | Average duration of sustained inactivity bouts during the waking hours on week days (WD) with a threshold of 5 minutes for both sleep and wakefulness (T5A5). |
| duration_sib_wakinghours_WD_T5A5_sd | Standard deviation of the duration of sustained inactivity bouts during the waking hours on week days (WD) with a threshold of 5 minutes for both sleep and wakefulness (T5A5). |
| number_sib_sleepperiod_WD_T5A5_mn | Average number of sustained inactivity bouts during the sleep period time window on week days (WD) with a threshold of 5 minutes for both sleep and wakefulness (T5A5). |
| number_sib_sleepperiod_WD_T5A5_sd | Standard deviation of the number of sustained inactivity bouts during the sleep period time window on week days (WD) with a threshold of 5 minutes for both sleep and wakefulness (T5A5). |
| number_sib_wakinghours_WD_T5A5_mn | Average number of sustained inactivity bouts during the waking hours on week days (WD) with a threshold of 5 minutes for both sleep and wakefulness (T5A5). |
| number_sib_wakinghours_WD_T5A5_sd | Standard deviation of the number of sustained inactivity bouts during the waking hours on week days (WD) with a threshold of 5 minutes for both sleep and wakefulness (T5A5). |
| duration_sib_wakinghours_atleast15min_WD_T5A5_mn | Mean duration of sustained inactivity bouts during waking hours on weekdays, with each bout lasting at least 15 minutes and within the acceleration thresholds specified for light activity. |
| duration_sib_wakinghours_atleast15min_WD_T5A5_sd | Standard deviation of the duration of sustained inactivity bouts during waking hours on weekdays, with each bout lasting at least 15 minutes and within the acceleration thresholds specified for light activity. |
| average_dur_sib_wakinghours_WD_T5A5_mn | Mean average duration of sustained inactivity bouts during waking hours on weekdays, within the acceleration thresholds specified for light activity. |
| average_dur_sib_wakinghours_WD_T5A5_sd | Standard deviation of the mean average duration of sustained inactivity bouts during waking hours on weekdays, within the acceleration thresholds specified for light activity. |
| n_days_w_sib_wakinghours_WD_T5A5 | Number of days with sustained inactivity bouts during waking hours on weekdays, within the acceleration thresholds specified for light activity. |
| sleeponset_WD_T5A5_mn | Mean sleep onset time on weekdays, within the acceleration thresholds specified for light activity. |
| sleeponset_WD_T5A5_sd | Standard deviation of the sleep onset time on weekdays, within the acceleration thresholds specified for light activity. |
| wakeup_WD_T5A5_mn | Mean wakeup time on weekdays, within the acceleration thresholds specified for light activity. |
| wakeup_WD_T5A5_sd | Standard deviation of the wakeup time on weekdays, within the acceleration thresholds specified for light activity. |
| SptDuration_WE_T5A5_mn | Mean Sleep Period Time duration on weekends, within the acceleration thresholds specified for light activity. |
| SptDuration_WE_T5A5_sd | Standard deviation of the Sleep Period Time duration on weekends, within the acceleration thresholds specified for light activity. |
| SleepDurationInSpt_WE_T5A5_mn | Mean total sleep duration within the Sleep Period Time on weekends, within the acceleration thresholds specified for light activity. |
| SleepDurationInSpt_WE_T5A5_sd | Standard deviation of the total sleep duration within the Sleep Period Time on weekends, within the acceleration thresholds specified for light activity. |
| sleep_efficiency_WE_T5A5_mn | Mean sleep efficiency within the Sleep Period Time on weekends, within the acceleration thresholds specified for light activity. |
| sleep_efficiency_WE_T5A5_sd | Standard deviation of sleep efficiency within the Sleep Period Time on weekends, within the acceleration thresholds specified for light activity. |
| duration_sib_wakinghours_WE_T5A5_mn | Mean duration of sustained inactivity bouts during waking hours on weekends, within the acceleration thresholds specified for light activity. |
| duration_sib_wakinghours_WE_T5A5_sd | Standard deviation of the duration of sustained inactivity bouts during waking hours on weekends, within the acceleration thresholds specified for light activity. |
| number_sib_sleepperiod_WE_T5A5_mn | Mean number of sustained inactivity bouts within the Sleep Period Time on weekends, within the acceleration thresholds specified for light activity. |
| number_sib_sleepperiod_WE_T5A5_sd | Standard deviation of the number of sustained inactivity bouts within the Sleep Period Time on weekends, within the acceleration thresholds specified for light activity. |
| number_sib_wakinghours_WE_T5A5_mn | Mean number of sustained inactivity bouts during waking hours on weekends, within the acceleration thresholds specified for light activity. |
| number_sib_wakinghours_WE_T5A5_sd | Standard deviation of the number of sustained inactivity bouts during waking hours on weekends, within the acceleration thresholds specified for light activity. |
| duration_sib_wakinghours_atleast15min_WE_T5A5_mn | Mean duration of sustained inactivity bouts during waking hours on weekends, with each bout lasting at least 15 minutes and within the acceleration thresholds specified for light activity. |
| duration_sib_wakinghours_atleast15min_WE_T5A5_sd | Standard deviation of the duration of sustained inactivity bouts during waking hours on weekends, with each bout lasting at least 15 minutes and within the acceleration thresholds specified for light activity. |
| average_dur_sib_wakinghours_WE_T5A5_mn | Mean average duration of sustained inactivity bouts during waking hours on weekends, within the acceleration thresholds specified for light activity. |
| average_dur_sib_wakinghours_WE_T5A5_sd | Standard deviation of the mean average duration of sustained inactivity bouts during waking hours on weekends, within the acceleration thresholds specified for light activity. |
| n_days_w_sib_wakinghours_WE_T5A5 | Number of days with sustained inactivity bouts during waking hours on weekends, using a threshold of 5 minutes for duration and 5 mg for acceleration. |
| sleeponset_WE_T5A5_mn | Mean sleep onset time on weekends with sustained inactivity bouts during waking hours using a threshold of 5 minutes for duration and 5 mg for acceleration. |
| sleeponset_WE_T5A5_sd | Standard deviation of sleep onset time on weekends with sustained inactivity bouts during waking hours using a threshold of 5 minutes for duration and 5 mg for acceleration. |
| wakeup_WE_T5A5_mn | Mean wakeup time on weekends with sustained inactivity bouts during waking hours using a threshold of 5 minutes for duration and 5 mg for acceleration. |
| wakeup_WE_T5A5_sd | Standard deviation of wakeup time on weekends with sustained inactivity bouts during waking hours using a threshold of 5 minutes for duration and 5 mg for acceleration. |
| sleeponset_pla | Plain average sleep onset time across all valid days. |
| wakeup_pla | Plain average wakeup time across all valid days. |
| sleeplog_used_pla | Plain average of whether a sleep log was used (TRUE) or not (FALSE) across all valid days. |
| nonwear_perc_day_pla | Plain average non-wear percentage during waking hours across all valid days. |
| nonwear_perc_spt_pla | Plain average non-wear percentage during Sleep Period Time across all valid days. |
| nonwear_perc_day_spt_pla | Plain average non-wear percentage during the whole day (including waking and SPT) across all valid days. |
| dur_spt_sleep_min_pla | Plain average duration of sleep during the Sleep Period Time across all valid days. |
| dur_spt_wake_IN_min_pla | Plain average duration of inactivity bouts during the SPT across all valid days. |
| dur_spt_wake_LIG_min_pla | Plain average duration of light activity bouts during the SPT across all valid days. |
| dur_spt_wake_MOD_min_pla | Plain average duration of moderate activity bouts during the SPT across all valid days. |
| dur_spt_wake_VIG_min_pla | Plain average duration of vigorous activity bouts during the SPT across all valid days. |
| dur_day_IN_unbt_min_pla | Plain average duration of unbouted inactivity during waking hours across all valid days. |
| dur_day_LIG_unbt_min_pla | Plain average duration of unbouted light activity during waking hours across all valid days. |
| dur_day_MOD_unbt_min_pla | Plain average duration of unbouted moderate activity during waking hours across all valid days. |
| dur_day_VIG_unbt_min_pla | Plain average duration of unbouted vigorous activity during waking hours across all valid days. |
| dur_day_MVPA_bts_1_min_pla | Plain average duration of bouted moderate-to-vigorous activity with bout duration of at least 1 minute during waking hours across all valid days. |
| dur_day_IN_bts_30_min_pla | Plain average duration of bouted inactivity with bout duration of 30 minutes or more during waking hours across all valid days. |
| dur_day_IN_bts_10_30_min_pla | Plain average duration of bouted inactivity with bout duration between 10 and 30 minutes during waking hours across all valid days. |
| dur_day_IN_bts_1_10_min_pla | Plain average duration of bouted inactivity with bout duration between 1 and 10 minutes during waking hours across all valid days. |
| dur_day_LIG_bts_10_min_pla | Plain average duration of bouted light activity with bout duration of at least 10 minutes during waking hours across all valid days. |
| dur_day_LIG_bts_1_10_min_pla | Plain average duration of bouted light activity with bout duration between 1 and 10 minutes during waking hours across all valid days. |
| dur_day_total_IN_min_pla | Plain average total duration of inactivity (bouted and unbouted) during waking hours across all valid days. |
| dur_day_total_LIG_min_pla | Plain average total duration of light activity (bouted and unbouted) during waking hours across all valid days. |
| dur_day_total_MOD_min_pla | Plain average total duration of moderate activity (bouted and unbouted) during waking hours across all valid days. |
| dur_day_total_VIG_min_pla | Plain average total duration of vigorous activity (bouted and unbouted) during waking hours across all valid days. |
| dur_day_min_pla | Plain average duration of waking hours across all valid days. |
| dur_spt_min_pla | Plain average duration of Sleep Period Time across all valid days. |
| dur_day_spt_min_pla | Plain average duration of the day (including both waking hours and SPT) across all valid days. |
| N_atleast5minwakenight_pla | Plain average number of nights with at least 5 minutes of wakefulness during sleep across all valid days. |
| sleep_efficiency_pla | Plain average sleep efficiency within the Sleep Period Time (SPT) window across all valid days. |
| ACC_spt_sleep_mg_pla | Plain average acceleration during sleep within the SPT window, measured in milligees (mg), across all valid days. |
| ACC_spt_wake_IN_mg_pla | Plain average acceleration during wakefulness within the SPT window categorized as inactivity (IN), measured in milligees (mg), across all valid days. |
| ACC_spt_wake_LIG_mg_pla | Plain average acceleration during wakefulness within the SPT window categorized as light activity (LIG), measured in milligees (mg), across all valid days. |
| ACC_spt_wake_MOD_mg_pla | Plain average acceleration during wakefulness within the SPT window categorized as moderate activity (MOD), measured in milligees (mg), across all valid days. |
| ACC_spt_wake_VIG_mg | Plain average acceleration during wakefulness within the SPT window categorized as vigorous activity (VIG), measured in milligees (mg), across all valid days. |
| ACC_day_IN_unbt_mg_pla | Plain average acceleration during daytime categorized as inactivity (IN) for unbouted behavior, measured in milligees (mg), across all valid days. |
| ACC_day_LIG_unbt_mg_pla | Plain average acceleration during daytime categorized as light activity (LIG) for unbouted behavior, measured in milligees (mg), across all valid days. |
| ACC_day_MOD_unbt_mg_pla | Plain average acceleration during daytime categorized as moderate activity (MOD) for unbouted behavior, measured in milligees (mg), across all valid days. |
| ACC_day_VIG_unbt_mg_pla | Plain average acceleration during daytime categorized as vigorous activity (VIG) for unbouted behavior, measured in milligees (mg), across all valid days. |
| ACC_day_MVPA_bts_1_mg_pla | Plain average acceleration during daytime categorized as moderate-to-vigorous physical activity (MVPA) for bouts lasting at least 1 minute, measured in milligees (mg), across all valid days. |
| ACC_day_IN_bts_30_mg_pla | Plain average acceleration during daytime categorized as inactivity (IN) for bouts lasting at least 30 minutes, measured in milligees (mg), across all valid days. |
| ACC_day_IN_bts_10_30_mg_pla | Plain average acceleration during daytime categorized as inactivity (IN) for bouts lasting at least 10 and less than 30 minutes, measured in milligees (mg), across all valid days. |
| ACC_day_IN_bts_1_10_mg_pla | Plain average acceleration during daytime categorized as inactivity (IN) for bouts lasting at least 1 and less than 10 minutes, measured in milligees (mg), across all valid days. |
| ACC_day_LIG_bts_10_mg_pla | Plain average acceleration during daytime categorized as light activity (LIG) for bouts lasting at least 10 minutes, measured in milligees (mg), across all valid days. |
| ACC_day_LIG_bts_1_10_mg_pla | Plain average acceleration during daytime categorized as light activity (LIG) for bouts lasting at least 1 and less than 10 minutes, measured in milligees (mg), across all valid days. |
| ACC_day_total_IN_mg_pla | Plain average total time spent in inactivity during daytime, measured in milligees (mg), across all valid days. |
| ACC_day_total_LIG_mg_pla | Plain average total time spent in light activity during daytime, measured in milligees (mg), across all valid days. |
| ACC_day_total_MOD_mg_pla | Plain average total time spent in moderate activity during daytime, measured in milligees (mg), across all valid days. |
| ACC_day_total_VIG_mg_pla | Plain average total time spent in vigorous activity during daytime, measured in milligees (mg), across all valid days. |
| ACC_day_mg_pla | Plain average acceleration during the entire daytime period, measured in milligees (mg), across all valid days. |
| ACC_spt_mg_pla | Plain average acceleration during the entire SPT window, measured in milligees (mg), across all valid days. |
| ACC_day_spt_mg_pla | Plain average acceleration during the combined duration of daytime and SPT, measured in milligees (mg), across all valid days. |
| quantile_mostactive60min_mg_pla | Plain average acceleration value for the 60-minute period with the highest activity level, measured in milligees (mg), across all valid days. |
| quantile_mostactive30min_mg_pla | Plain average acceleration value for the 30-minute period with the highest activity level, measured in milligees (mg), across all valid days. |
| L5VALUE_pla | Plain average of the acceleration value during the least active five hours in a day. |
| M5VALUE_pla | Plain average of the acceleration value during the most active five hours in a day. |
| L5TIME_num_pla | Plain average of the timing of the least active five hours in numeric format (hours after the previous midnight). |
| M5TIME_num_pla | Plain average of the timing of the most active five hours in numeric format (hours after the previous midnight). |
| Nbouts_day_MVPA_bts_1_pla | Plain average of the number of bouts of moderate-to-vigorous physical activity (MVPA) lasting at least 1 minute during the waking hours. |
| Nbouts_day_IN_bts_30_pla | Plain average of the number of inactivity bouts lasting at least 30 minutes during the waking hours. |
| Nbouts_day_IN_bts_10_30_pla | Plain average of the number of inactivity bouts lasting between 10 and 30 minutes during the waking hours. |
| Nbouts_day_IN_bts_1_10_pla | Plain average of the number of inactivity bouts lasting between 1 and 10 minutes during the waking hours. |
| Nbouts_day_LIG_bts_10_pla | Plain average of the number of light activity bouts lasting at least 10 minutes during the waking hours. |
| Nbouts_day_LIG_bts_1_10_pla | Plain average of the number of light activity bouts lasting between 1 and 10 minutes during the waking hours. |
| Nblocks_spt_sleep_pla | Plain average of the number of sleep blocks during the Sleep Period Time. |
| Nblocks_spt_wake_IN_pla | Plain average of the number of wakefulness blocks classified as inactivity during the Sleep Period Time. |
| Nblocks_spt_wake_LIG_pla | Plain average of the number of wakefulness blocks classified as light activity during the Sleep Period Time. |
| Nblocks_spt_wake_MOD_pla | Plain average of the number of wakefulness blocks classified as moderate activity during the Sleep Period Time. |
| Nblocks_spt_wake_VIG_pla | Plain average of the number of wakefulness blocks classified as vigorous activity during the Sleep Period Time. |
| Nblocks_day_IN_unbt_pla | Plain average of the number of inactivity blocks (unbouted) during the waking hours. |
| Nblocks_day_LIG_unbt_pla | Plain average of the number of light activity blocks (unbouted) during the waking hours. |
| Nblocks_day_MOD_unbt_pla | Plain average of the number of moderate activity blocks (unbouted) during the waking hours. |
| Nblocks_day_VIG_unbt_pla | Plain average of the number of vigorous activity blocks (unbouted) during the waking hours. |
| Nblocks_day_MVPA_bts_1_pla | Plain average of the number of MVPA bouts (bouted) lasting at least 1 minute during the waking hours. |
| Nblocks_day_IN_bts_30_pla | Plain average of the number of inactivity bouts (bouted) lasting at least 30 minutes during the waking hours. |
| Nblocks_day_IN_bts_10_30_pla | Plain average of the number of inactivity bouts (bouted) lasting between 10 and 30 minutes during the waking hours. |
| Nblocks_day_IN_bts_1_10_pla | Plain average of the number of inactivity bouts (bouted) lasting between 1 and 10 minutes during the waking hours. |
| Nblocks_day_LIG_bts_10_pla | Plain average of the number of light activity bouts (bouted) lasting at least 10 minutes during the waking hours. |
| Nblocks_day_LIG_bts_1_10_pla | Plain average of the number of light activity bouts (bouted) lasting between 1 and 10 minutes during the waking hours. |
| Nblocks_day_total_IN_pla | Plain average of the total number of inactivity blocks (both bouted and unbouted) during the waking hours. |
| Nblocks_day_total_LIG_pla | Plain average of the total number of light activity blocks (both bouted and unbouted) during the waking hours. |
| Nblocks_day_total_MOD_pla | Plain average of the total number of moderate activity blocks (both bouted and unbouted) during the waking hours. |
| Nblocks_day_total_VIG_pla | Plain average of the total number of vigorous activity blocks (both bouted and unbouted) during the waking hours. |
| boutcriter.in | Bout criterion for inactivity. |
| boutcriter.lig | Bout criterion for light activity. |
| boutcriter.mvpa | Bout criterion for moderate-to-vigorous physical activity. |
| boutdur.in | Bout duration for inactivity. |
| boutdur.lig | Bout duration for light activity. |
| boutdur.mvpa_pla | Bout duration for moderate-to-vigorous physical activity, plain average. |
| bout.metric | Metric used for defining activity bouts. |
| sleeponset_wei | Weighted average of sleep onset time. |
| wakeup_wei | Weighted average of wakeup time. |
| sleeplog_used_wei | Weighted average of whether a sleep log was used. |
| nonwear_perc_day_wei | Weighted average of non-wear percentage during waking hours. |
| nonwear_perc_spt_wei | Weighted average of non-wear percentage during sleep period time. |
| nonwear_perc_day_spt_wei | Weighted average of non-wear percentage during the entire day. |
| dur_spt_sleep_min_wei | Weighted average of sleep duration during sleep period time. |
| dur_spt_wake_IN_min_wei | Weighted average of wake duration during sleep period time for inactivity. |
| dur_spt_wake_LIG_min_wei | Weighted average of wake duration during sleep period time for light activity. |
| dur_spt_wake_MOD_min_wei | Weighted average of wake duration during sleep period time for moderate activity. |
| dur_spt_wake_VIG_min_wei | Weighted average of wake duration during sleep period time for vigorous activity. |
| dur_day_IN_unbt_min_wei | Weighted average of inactivity duration during the day, unbouted. |
| dur_day_LIG_unbt_min_wei | Weighted average of light activity duration during the day, unbouted. |
| dur_day_MOD_unbt_min_wei | Weighted average of moderate activity duration during the day, unbouted. |
| dur_day_VIG_unbt_min_wei | Weighted average of vigorous activity duration during the day, unbouted. |
| dur_day_MVPA_bts_1_min_wei | Weighted average of moderate-to-vigorous physical activity duration during the day, bouted (bout duration 1 minute). |
| dur_day_IN_bts_30_min_wei | Weighted average of inactivity duration during the day, bouted (bout duration 30 minutes). |
| dur_day_IN_bts_10_30_min_wei | Weighted average of inactivity duration during the day, bouted (bout duration between 10 and 30 minutes). |
| dur_day_IN_bts_1_10_min_wei | Weighted average of inactivity duration during the day, bouted (bout duration between 1 and 10 minutes). |
| dur_day_LIG_bts_10_min_wei | Weighted average of light activity duration during the day, bouted (bout duration 10 minutes). |
| dur_day_LIG_bts_1_10_min_wei | Weighted average of light activity duration during the day, bouted (bout duration between 1 and 10 minutes). |
| dur_day_total_IN_min_wei | Weighted average of total inactivity duration during the day. |
| dur_day_total_LIG_min_wei | Weighted average of total light activity duration during the day. |
| dur_day_total_MOD_min_wei | Weighted average of total moderate activity duration during the day. |
| dur_day_total_VIG_min_wei | Weighted average of total vigorous activity duration during the day. |
| dur_day_min_wei | Weighted average of total duration during the day. |
| dur_spt_min_wei | Weighted average of total duration during sleep period time. |
| dur_day_spt_min_wei | Weighted average of total duration during the entire day (including both waking and sleep period time). |
| N_atleast5minwakenight_wei | Weighted average of the number of nights with at least 5 minutes of wakefulness. |
| sleep_efficiency_wei | Weighted average of sleep efficiency. |
| ACC_spt_sleep_mg_wei | Weighted average of accelerometer-derived sleep efficiency during sleep period time. |
| ACC_spt_wake_IN_mg_wei | Weighted average of accelerometer-derived inactivity duration during sleep period time. |
| ACC_spt_wake_LIG_mg_wei | Weighted average of accelerometer-derived light activity duration during sleep period time. |
| ACC_spt_wake_MOD_mg_wei | Weighted average of accelerometer-derived moderate activity duration during sleep period time. |
| ACC_day_IN_unbt_mg_wei | Weighted average of accelerometer-derived inactivity duration during the day, unbouted. |
| ACC_day_LIG_unbt_mg_wei | Weighted average of accelerometer-derived light activity duration during the day, unbouted. |
| ACC_day_MOD_unbt_mg_wei | Weighted average of accelerometer-derived moderate activity duration during the day, unbouted. |
| ACC_day_VIG_unbt_mg_wei | Weighted average of accelerometer-derived vigorous activity duration during the day, unbouted. |
| ACC_day_MVPA_bts_1_mg_wei | Weighted average of accelerometer-derived moderate-to-vigorous physical activity duration during the day, bouted (bout duration 1 minute). |
| ACC_day_IN_bts_30_mg_wei | Weighted average of accelerometer-derived inactivity duration during the day, bouted (bout duration 30 minutes). |
| ACC_day_IN_bts_10_30_mg_wei | Weighted average of accelerometer-derived inactivity duration during the day, bouted (bout duration between 10 and 30 minutes). |
| ACC_day_IN_bts_1_10_mg_wei | Weighted average of accelerometer-derived inactivity duration during the day, bouted (bout duration between 1 and 10 minutes). |
| ACC_day_LIG_bts_10_mg_wei | Weighted average of accelerometer-derived light activity duration during the day, bouted (bout duration 10 minutes). |
| ACC_day_LIG_bts_1_10_mg_wei | Weighted average of accelerometer-derived light activity duration during the day, bouted (bout duration between 1 and 10 minutes). |
| ACC_day_total_IN_mg_wei | Weighted average of accelerometer-derived total inactivity duration during the day. |
| ACC_day_total_LIG_mg_wei | Weighted average of accelerometer-derived total light activity duration during the day. |
| ACC_day_total_MOD_mg_wei | Weighted average of accelerometer-derived total moderate activity duration during the day. |
| ACC_day_total_VIG_mg_wei | Weighted average of accelerometer-derived total vigorous activity duration during the day. |
| ACC_day_mg_wei | Weighted average of accelerometer-derived total activity duration during the day. |
| ACC_spt_mg_wei | Weighted average of accelerometer-derived total activity duration during sleep period time. |
| ACC_day_spt_mg_wei | Weighted average of accelerometer-derived total activity duration during the entire day (including both waking and sleep period time). |
| quantile_mostactive60min_mg_wei | Weighted average of the accelerometer-derived quantile for the most active 60 minutes. |
| quantile_mostactive30min_mg_wei | Weighted average of the accelerometer-derived quantile for the most active 30 minutes. |
| L5VALUE_wei | Weighted average of acceleration value during the least active five hours. |
| M5VALUE_wei | Weighted average of acceleration value during the most active five hours. |
| L5TIME_num_wei | Weighted average of timing of the least active five hours in numeric format (hours after the previous midnight). |
| M5TIME_num_wei | Weighted average of timing of the most active five hours in numeric format (hours after the previous midnight). |
| Nbouts_day_MVPA_bts_1_wei | Weighted average of the number of MVPA bouts (bouted) lasting at least 1 minute during the waking hours. |
| Nbouts_day_IN_bts_30_wei | Weighted average of the number of inactivity bouts (bouted) lasting at least 30 minutes during the waking hours. |
| Nbouts_day_IN_bts_10_30_wei | Weighted average of the number of inactivity bouts (bouted) lasting between 10 and 30 minutes during the waking hours. |
| Nbouts_day_IN_bts_1_10_wei | Weighted average of the number of inactivity bouts (bouted) lasting between 1 and 10 minutes during the waking hours. |
| Nbouts_day_LIG_bts_10_wei | Weighted average of the number of light activity bouts (bouted) lasting at least 10 minutes during the waking hours. |
| Nbouts_day_LIG_bts_1_10_wei | Weighted average of the number of light activity bouts (bouted) lasting between 1 and 10 minutes during the waking hours. |
| Nblocks_spt_sleep_wei | Weighted average of the number of sleep blocks during the Sleep Period Time. |
| Nblocks_spt_wake_IN_wei | Weighted average of the number of wakefulness blocks classified as inactivity during the Sleep Period Time. |
| Nblocks_spt_wake_LIG_wei | Weighted average of the number of wakefulness blocks classified as light activity during the Sleep Period Time. |
| Nblocks_spt_wake_MOD_wei | Weighted average of the number of wakefulness blocks classified as moderate activity during the Sleep Period Time. |
| Nblocks_spt_wake_VIG_wei | Weighted average of the number of wakefulness blocks classified as vigorous activity during the Sleep Period Time. |
| Nblocks_day_IN_unbt_wei | Weighted average of the number of inactivity blocks (unbouted) during the waking hours. |
| Nblocks_day_LIG_unbt_wei | Weighted average of the number of light activity blocks (unbouted) during the waking hours. |
| Nblocks_day_MOD_unbt_wei | Weighted average of the number of moderate activity blocks (unbouted) during the waking hours. |
| Nblocks_day_VIG_unbt_wei | Weighted average of the number of vigorous activity blocks (unbouted) during the waking hours. |
| Nblocks_day_MVPA_bts_1_wei | Weighted average of the number of MVPA bouts (bouted) lasting at least 1 minute during the waking hours. |
| Nblocks_day_IN_bts_30_wei | Weighted average of the number of inactivity bouts (bouted) lasting at least 30 minutes during the waking hours. |
| Nblocks_day_IN_bts_10_30_wei | Weighted average of the number of inactivity bouts (bouted) lasting between 10 and 30 minutes during the waking hours. |
| Nblocks_day_IN_bts_1_10_wei | Weighted average of the number of inactivity bouts (bouted) lasting between 1 and 10 minutes during the waking hours. |
| Nblocks_day_LIG_bts_10_wei | Weighted average of the number of light activity bouts (bouted) lasting at least 10 minutes during the waking hours. |
| Nblocks_day_LIG_bts_1_10_wei | Weighted average of the number of light activity bouts (bouted) lasting between 1 and 10 minutes during the waking hours. |
| Nblocks_day_total_IN_wei | Weighted average of the total number of inactivity blocks (both bouted and unbouted) during the waking hours. |
| Nblocks_day_total_LIG_wei | Weighted average of the total number of light activity blocks (both bouted and unbouted) during the waking hours. |
| Nblocks_day_total_MOD_wei | Weighted average of the total number of moderate activity blocks (both bouted and unbouted) during the waking hours. |
| Nblocks_day_total_VIG_wei | Weighted average of the total number of vigorous activity blocks (both bouted and unbouted) during the waking hours. |
