# Supplementary material for: Association between wrist-worn free-living accelerometry and hand grip strength in middle-aged and older adults
Source: Aging Clin Exp Res. 2024 May 8;36(1):108. doi: 10.1007/s40520-024-02757-z (PMC11078825; doi:10.1007/s40520-024-02757-z)

**
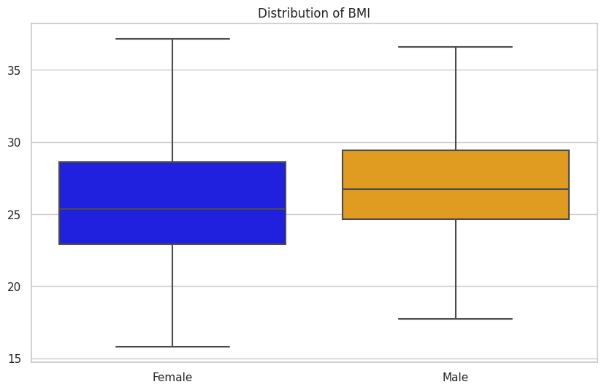
**
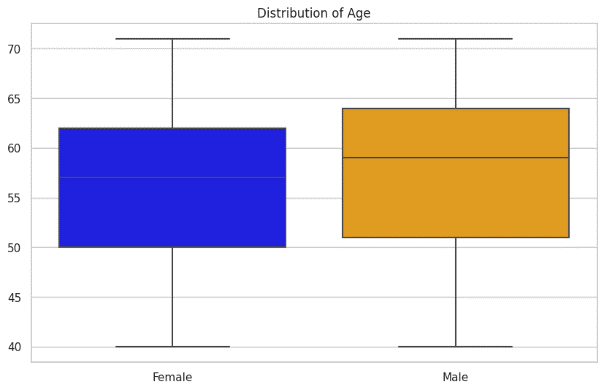
**
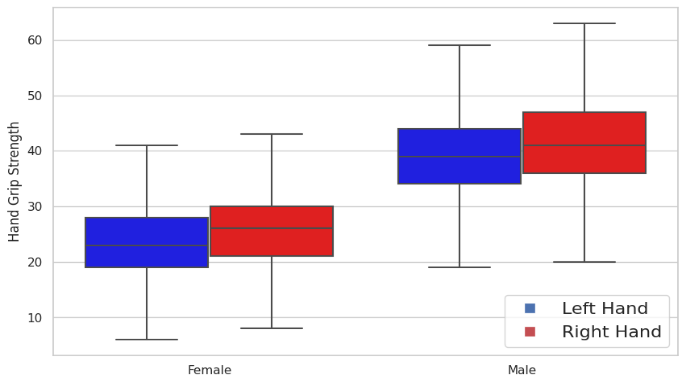
**

Figure A.1. Data distributions for both genders for hand grip strength, age, and BMI (N=14,161)


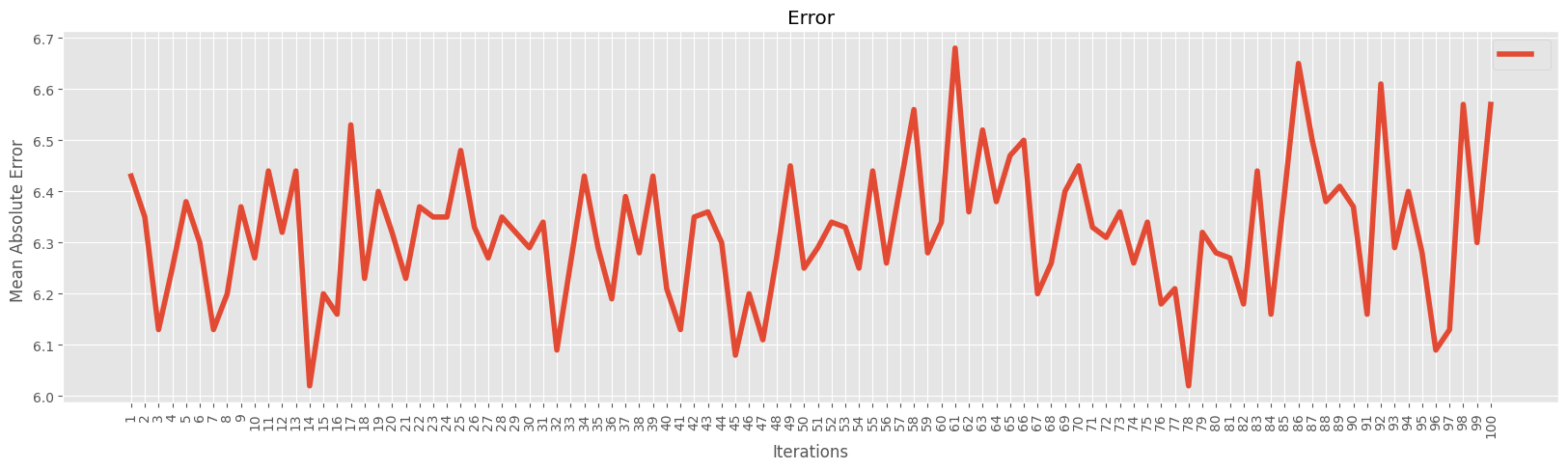

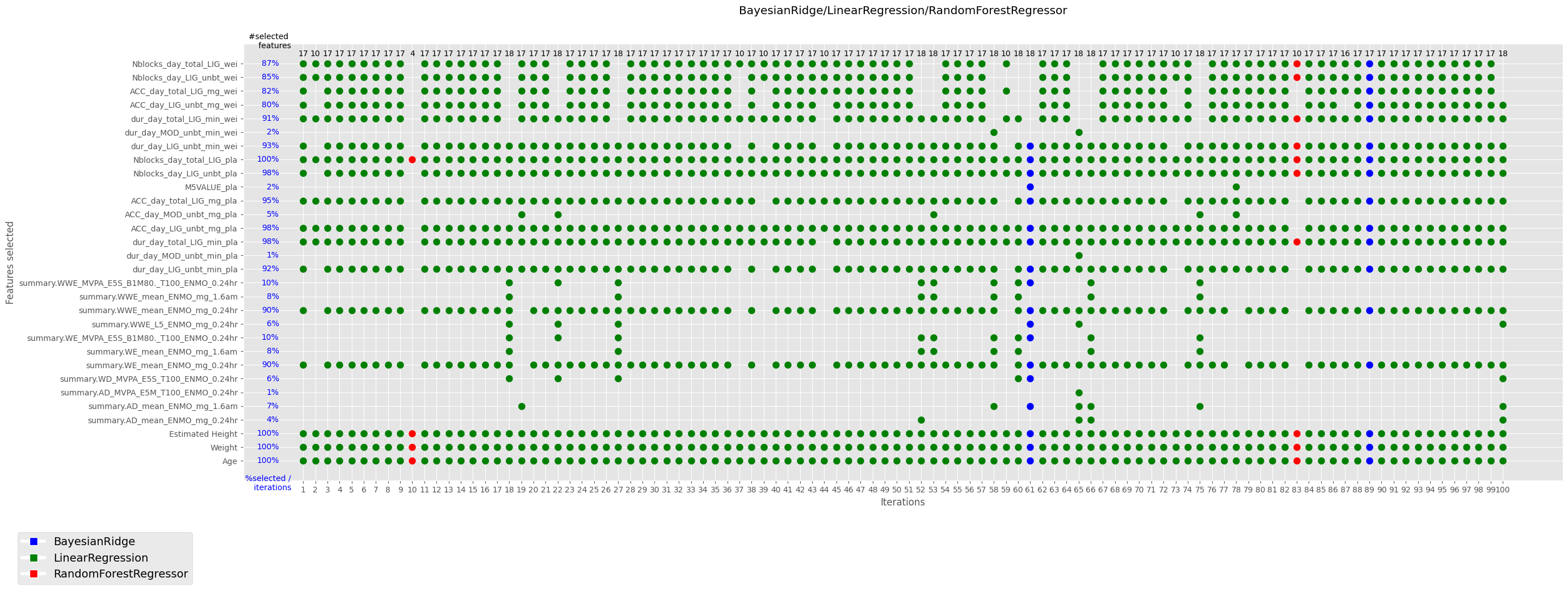


Figure A.2. MAE and hand grip strength parameters selected over all iterations (male)


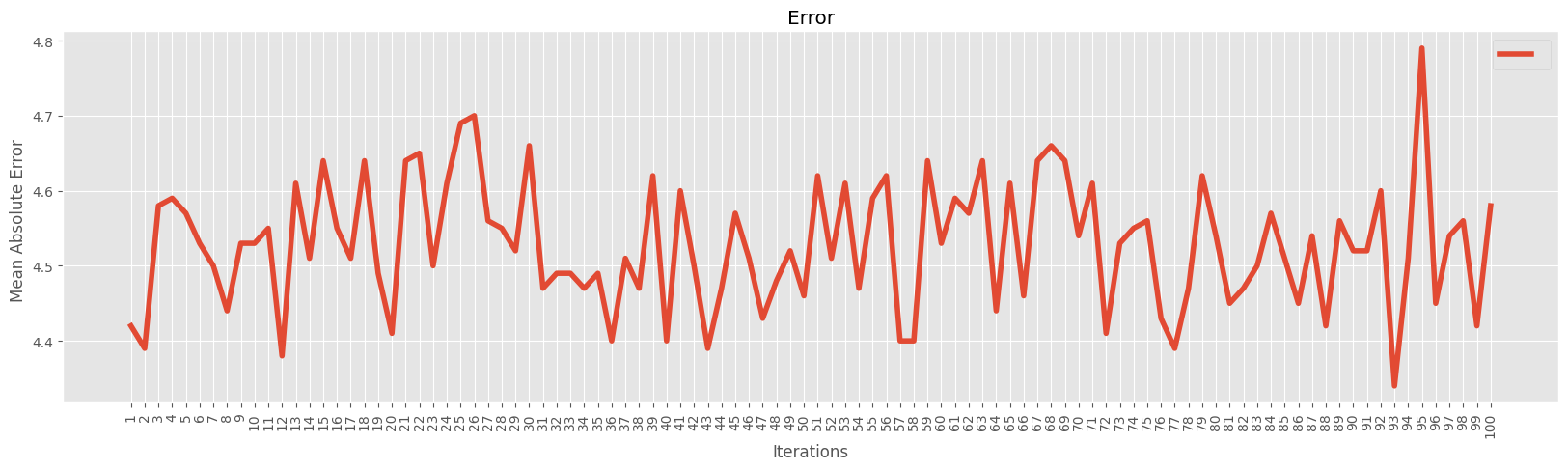

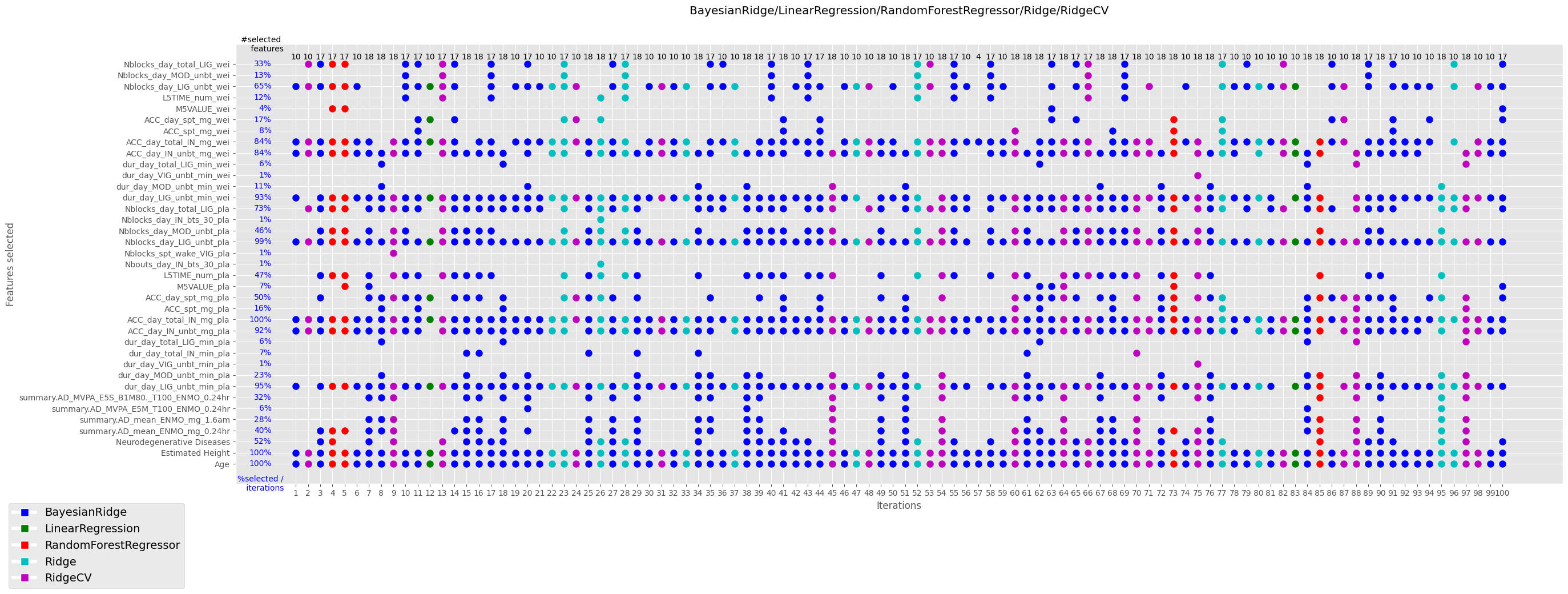


Figure A.3. MAE and hand grip strength parameters selected over all iterations (female)

Figure A.4: ROC curves for frailty classification of males using a T-score of -2 (top left), males using a T-score of -1 (top right), females using a T-score of -2 (bottom left), and females using a T-score of -1 (bottom right)


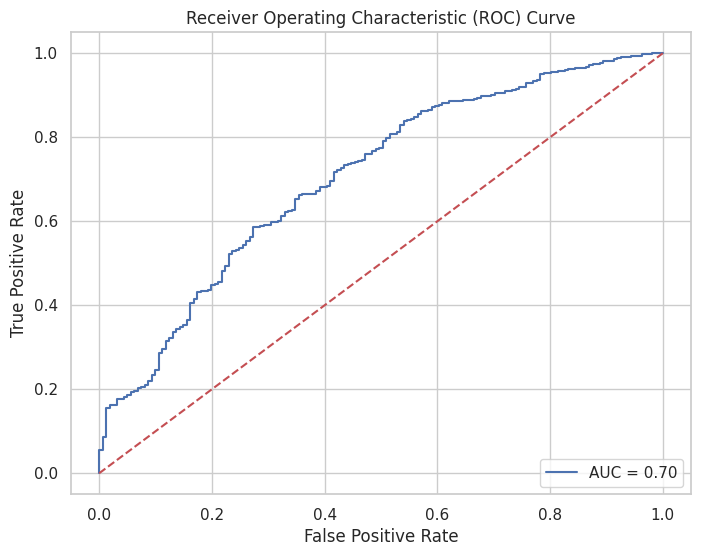

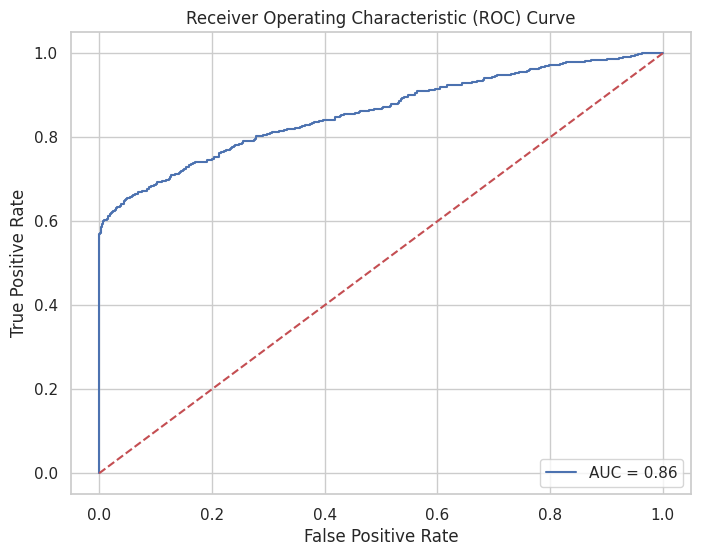

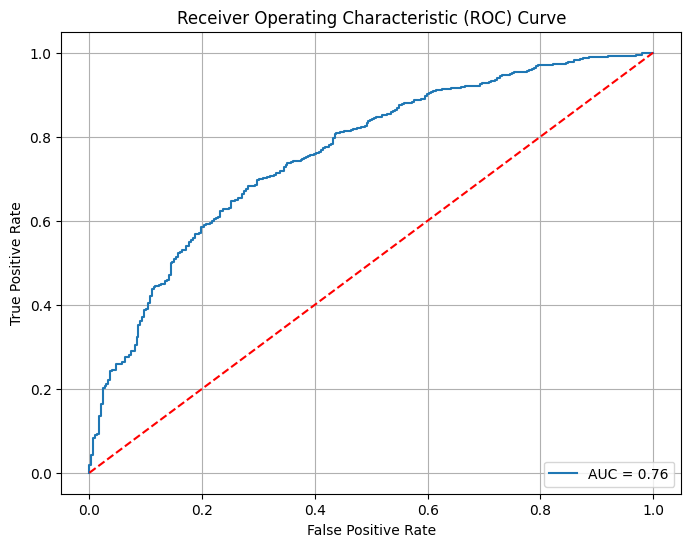

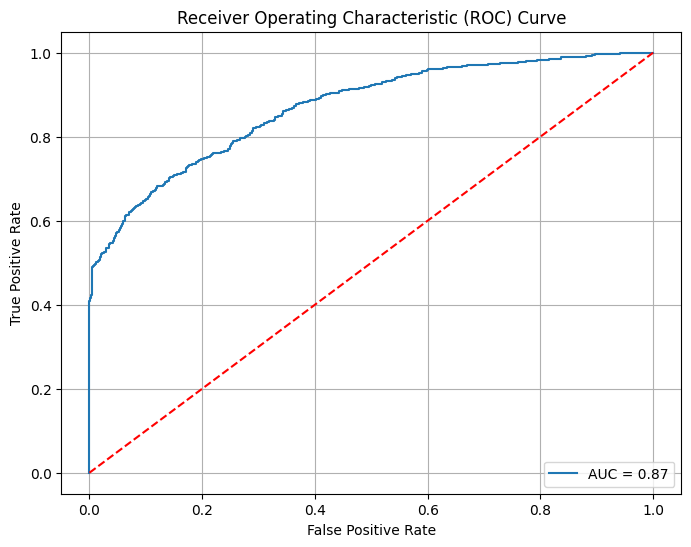

Supplement: Supplementary file 3 — Supplementary Material 3: Appendix 3. Figure A.1. Data distributions for both genders for hand grip strength, age, and BMI (N = 14,161). Figure A.2. MAE and hand grip strength parameters selected over all iterations (male). Figure A.3. MAE and hand grip strength parameters selected over all iterations (female). Figure A.4. ROC curves for frailty classification of males using a T-score of -2 (top left), males using a T-score of -1 (top right), females using a T-score of -2 (bottom left), and females using a T-score of -1 (bottom right). [file 40520_2024_2757_MOESM3_ESM.docx]
